# Crystal structures of 5,12-dimethyl-1,4,8,11-tetra­aza­cyclo­tetra­decane cobalt(III) mono-phenyl­acetyl­ide and bis-phenyl­acetyl­ide

**DOI:** 10.1107/S2056989018003997

**Published:** 2018-03-13

**Authors:** Benjamin M. Oxley, Brandon Mash, Matthias Zeller, Susannah Banziger, Tong Ren

**Affiliations:** a Department of Chemistry, Purdue University, 560 Oval Dr., W. Lafayette, IN 47907-2084, USA

**Keywords:** crystal structure, cobalt, acetyl­ide, DMC, cyclam

## Abstract

Reported in this contribution are the synthesis and crystal structures of new mono- and bis-phenyl­acetyl­ides based on Co^III^(DMC) (DMC is 5,12-dimethyl-1,4,8,11-tetra­aza­cyclo­tetra­deca­ne). Chlorido­(5,12-dimethyl-1,4,8,11-tetra­aza­cyclo­tetra­deca­ne)(phenyl­ethyn­yl)cobalt(III) chloride–aceto­nitrile–methanol (1/1/1), [Co(C_8_H_5_)Cl(C_12_H_28_N_4_)]Cl·CH_3_CN·CH_3_OH, **1**, and (5,12-dimethyl-1,4,8,11-tetra­aza­cyclo­tetra­deca­ne)bis­(phenyl­ethyn­yl)cobalt(III) tri­fluoro­methane­sulfonate–di­chloro­methane (2/1), [Co(C_8_H_5_)_2_(C_12_H_28_N_4_)]_2_(CF_3_SO_3_)_2_·CH_2_Cl_2_, **2**, were prepared under weak-base conditions in satisfactory yields. Single-crystal X-ray diffraction studies revealed that both **1** and **2** adopt a pseudo-octa­hedral symmetry in which the Cl—Co—C angles of **1** and C—Co—C of **2** range from 177.7 (2) to 178.0 (2)° and from 177.67 (9) to 179.67 (9)°, respectively. In both structures, the Co^III^ metal center is coordinated in the equatorial plane by four N atoms, in which the N—Co—N angles range from 85.6 (3) to 94.4 (3)°. The structure of **1** features two crystallographically independent mol­ecules in its triclinic cell (*Z*′ = 2), which are related to each other by pseudo-monoclinic symmetry. The crystal investigated was twinned by a symmetry operator of the approximate double-volume *C*-centered cell (180° rotation around [201] of the actual triclinic cell), with a refined twin ratio of 0.798 (3) to 0.202 (3). Both methanol solvent mol­ecules in **1** are disordered, the major occupancy rates refined to 0.643 (16) and 0.357 (16). Compound **2** also contains two mol­ecules in the asymmetric unit, together with two tri­fluoro­methane­sulfonate anions [of which one is disordered; occupancy values of 0.503 (16) and 0.497 (16)] and a disordered di­chloro­methane [occupancy values of 0.545 (12) and 0.455 (12)].

## Abstract 2

The crystal structures presented herein consist of two positively charged Co^III^(DMC) acetyl­ide complexes that take on a pseudo-octa­hedral symmetry and can be synthesized under weak-base conditions.

## Chemical context   

Alkynyl complexes of 3*d* metals supported by cyclam (1,4,8,11-tetra­aza­cyclo­tetra­deca­ne) and its *C*-functionalized derivatives have received intense attention in recent years (Ren, 2016 ▸). Inter­esting examples include magnetic couplings between Cr(cyclam) species mediated by ethynyl­tetra­thia­fulvalene ligands (Nishijo *et al.*, 2011 ▸), formation of Co^III^(cyclam) dimers and trimers through 1,4-diethynyl­benzene and 1,3,5-triethynyl­benzene bridges, respectively (Hoffert *et al.*, 2012 ▸), and phospho­rescence from [Cr(cyclam’)(C­_2_ *R*)_2_] type complexes with cyclam’ as either 5,5,7,12,12,14-hexa­methyl-1,4,8,11-tetra­aza­cyclo­tetra­decane (HMC; Tyler *et al.*, 2016 ▸) or 5,12-dimethyl-1,4,8,11-tetra­aza­cyclo­tetra­decane (DMC; Judkins *et al.*, 2017 ▸). A number of Co^III^-containing species have been elaborated in our laboratories, including the series [Co(cyclam)Cl]_2_(*m*-C_2m_) (*m* = 2 – 6; Cook *et al.*, 2015 ▸, 2016 ▸), the species containing cross-conjugated *gem*-DEE ligands (Natoli *et al.*, 2015 ▸, 2016 ▸), and the unsymmetric *trans-*[Co(cyclam)(C_2_Ar)(C_2_Ar’)] type complexes (Banziger *et al.*, 2015 ▸). Described in this contribution are the structural characterization of [Co^III^(DMC)(C_2_Ph)Cl]Cl (**1**) and [Co^III^(DMC)(C_2_Ph)_2_]OTf (**2**), which were prepared from [Co^III^(DMC)Cl_2_]Cl under weak-base conditions.
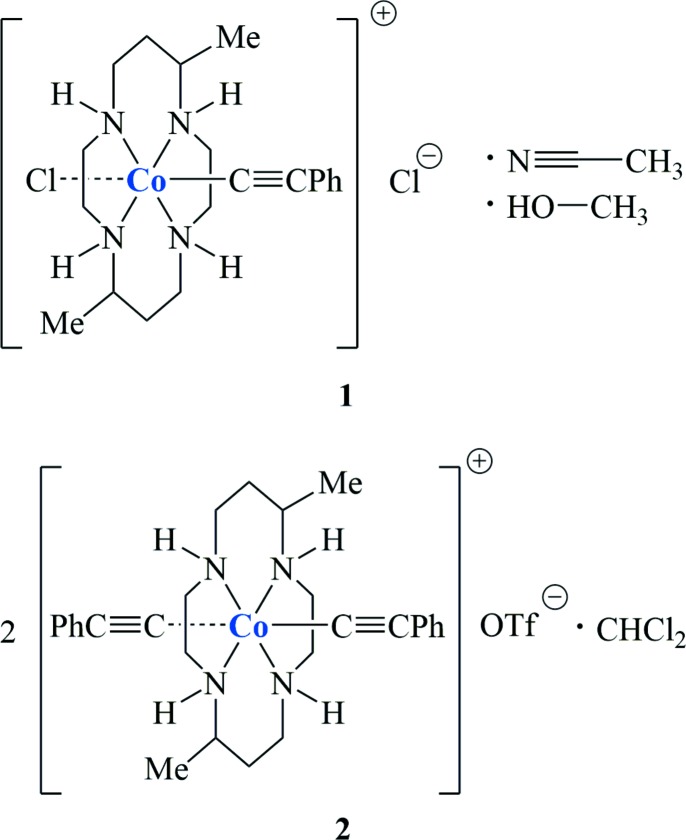


## Structural commentary   

**Figure 1:**
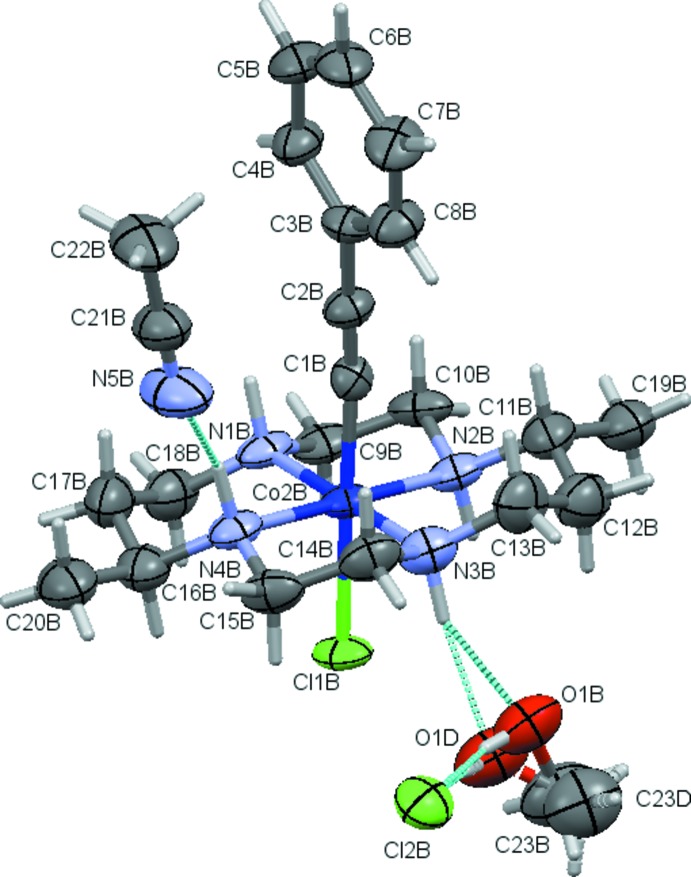
Displacement ellipsoid plot (50% probability setting) for one of the two pseudo-symmetry-related halves of the asymmetric unit of compound **1**, showing the atom-naming scheme and some of the hydrogen-bonding inter­actions (turquoise dashed lines). Shown is the ‘*B*-moiety’, the atom-naming scheme for the ‘*A*-moiety’ is equivalent. Labels for H atoms are omitted for clarity.

Compound **1** crystallizes in *P* 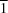 with two crystallographically independent moieties, Fig. 1 ▸. Each moiety consists of one complex [Co^III^(DMC)(C_2_Ph)(Cl)]^+^ cation, a chloride counter-ion, and one aceto­nitrile and methanol solvate mol­ecule, for a total composition of C_20_H_33_ClCoN_4_·C_2_H_3_N·CH_4_O·Cl. The two unique moieties, labeled *A* and *B*, are related by a pseudo-glide plane (see the *Supra­molecular features* section for a more detailed discussion), and a common atom-naming scheme was used for the contents of the two unique halves of the structure. Both methanol mol­ecules are disordered, with a common refined occupancy ratio of 0.643 (16):0.357 (16).

**Figure 2:**
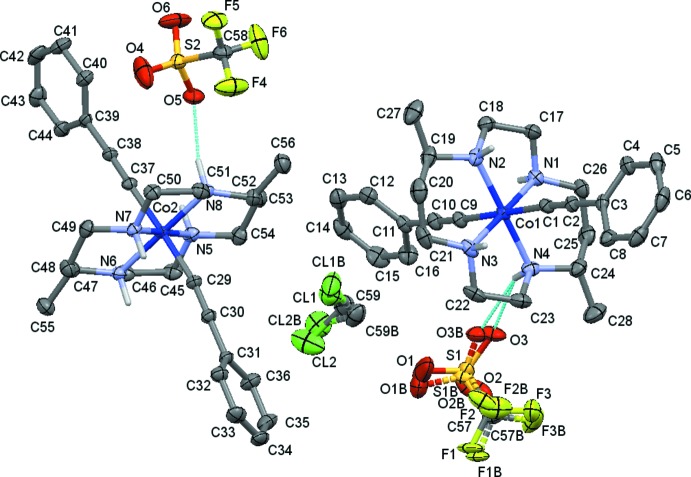
Displacement ellipsoid plot (50% probability setting) for compound **2**, showing the atom-naming scheme and some of the hydrogen-bonding inter­actions (turquoise dashed lines). The OTf molecule comprising S2 is in position *x* − 1, *y*, *z*. The carbon-bound H atoms and H-atom labels are omitted for clarity.

Compound **2** crystallizes in *P*2_1_, Fig. 2 ▸. Similar to **1**, **2** also features two unique cations and anions in its asymmetric unit, but they are not related by any crystallographic pseudo-symmetry. Each complex cation [Co^III^(DMC)(C_2_Ph)_2_]^+^ is paired with a triflate anion. The asymmetric unit is completed by a single methyl­ene chloride solvate mol­ecule, yielding a formula of 2(C_28_H_38_CoN_4_)·2(CF_3_O_3_S)·CH_2_Cl_2_. One of the triflate anions as well as the methyl­ene chloride mol­ecule were refined as disordered, with occupancy rates of 0.503 (22) and 0.545 (12) for the major components.

The mol­ecular geometries of the cations in **1** and **2** are similar (Tables 1 ▸ and 2 ▸). Both structures feature a central cobalt(III) ion with a pseudo-octa­hedral geometry. The metal ion is coordinated in the equatorial plane by the four amine nitro­gen atoms of a 5,12-dimethyl-1,4,8,11-tetra­aza­cyclo­tetra­decane (DMC) ligand. For compounds **1** and **2** respect­ively, the nearly linear C—Co—Cl [177.7 (2) and 178.0 (2)°] and C—Co—C [177.67 (9) and 179.67 (9)°] units are close to normal to the equatorial plane created by the coordinated amines of the macrocyclic ligand, confirming octa­hedral geometries. The C—Co—N and Cl—Co—N angles are all tightly clustered around 90°. The actual values range from 87.1 (1) to 92.9 (1)° (Tables 1 ▸ and 2 ▸). The N—Co—N angles are more variable, caused by the difference in size of the ethyl­ene and 1-methyl-propyl­ene bridges of the DMC ligand. They range from 85.6 (3) to 94.4 (3)°, with the smaller values being associated with the shorter ethyl­ene N–CH_2_–CH_2_–N chelates, and the larger with the wider N–CH(CH_3_)–CH_2_–CH_2_–N connections (Tables 1 ▸ and 2 ▸).

Some of the Co—C≡C angles deviate from perfect linearity, likely due to steric forces resulting from packing effects. The values range from 171.3 (7) to 174.2 (2)°, with the latter extreme value belonging to one of the Co—C≡C units of **2**. All Co—C≡C angles are given in Tables 1 ▸ and 2 ▸. Each macrocycle exhibits a *trans*-III RRSS conformation, characterized by two neighboring N—H amine units pointing upwards, while their two *trans* N—H counterparts point in the opposing direction. Other conformations, such as *trans*-I, II, IV, or *cis* conformations, are much less prevalent for both the DMC and other cyclam ligands when coordinated to transition metals. (Bosnich *et al.*, 1965 ▸; Hoffert *et al.*, 2012 ▸; Cook *et al.*, 2016 ▸).

The Co—C bond lengths [1.893 (7) and 1.905 (7) Å] for compound **1** are as expected for this class of compounds and compare well to values observed by Shores for the cyclam macrocyclic counterpart of **1**. (Hoffert *et al.*, 2012 ▸) Compound **2** shows characteristics of a trans-influence with elongated Co—C bond lengths [1.927 (2) Å avg.] relative to compound **1**. This effect is a result of the stronger π-donation from phenyl­acetyl­ide compared to chloride. The C—C and C≡C bond lengths of the phenyl­acetyl­ene ligands fall in the expected region for single and triple bonds respectively. The acetyl­ides in compound **2** show a slightly cumulenic character with elongated C≡C and shortened C—C bond lengths with respect to compound **1**, as was also seen by Shores and coworkers (Hoffert *et al.*, 2012 ▸). The Co—N bond lengths for each compound are presented in Tables 1 ▸ and 2 ▸ and do not deviate significantly from those in previously reported Co tetra­aza­macrocyclic compounds.

## Supra­molecular features   

**Figure 3:**
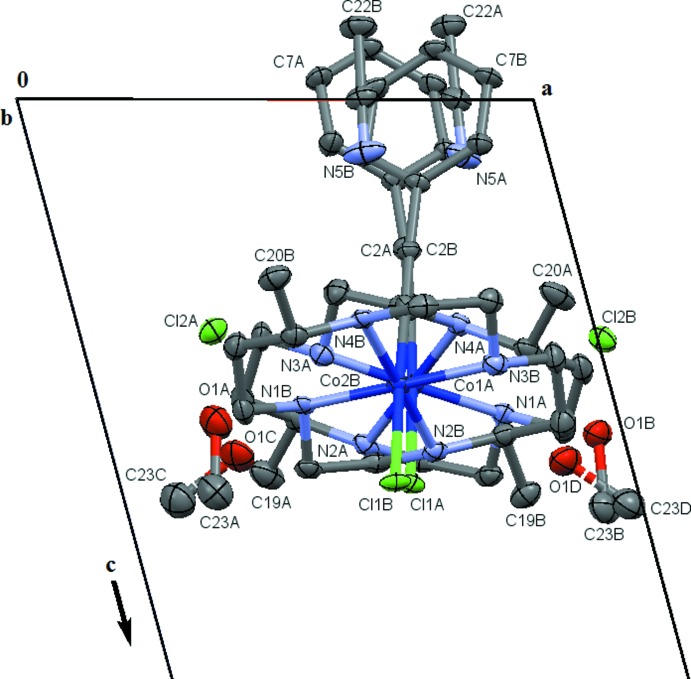
Displacement ellipsoid plot (set to a 20% probability setting for clarity) for compound **1**, showing the pseudo-glide plane perpendicular to the *a* axis. The shift direction is along **b**.

**Figure 4:**
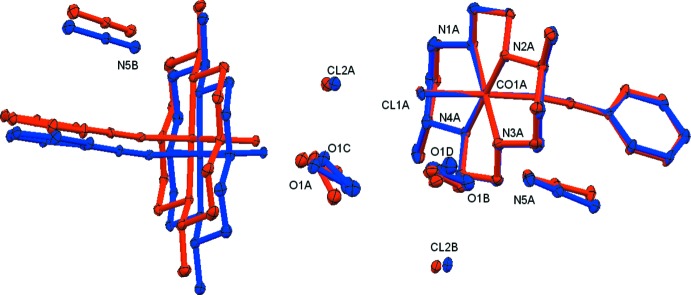
Least-squares overlay of one set of cations *A* and *B* (to the right) of compound **1**. Also shown are the surrounding chloride anions and solvate mol­ecules and a second cation (on the left). Atoms color coded red belong to the original structure and atoms in blue were inverted prior to the least-squares overlay. The least-squares fit is based on all atoms of the cation pair on the right (r.m.s. deviation = 0.138 Å). For this pair, labels are shown only for the *A* cation. Labels for atoms of the second pair of cations and for all carbon atoms are omitted for clarity.

The structure of the chlorine salt exhibits monoclinic pseudo-symmetry, emulating a double-volume *C*-centered unit cell with parameters *a* = 34.721, *b* = 9.690, *c* = 15.668 Å, and β = 93.41°. The α and γ angles in the monoclinic cell deviate substanti­ally from 90°, being 88.97 and 89.52° when not constrained during data integration. In the crystal structure, the monoclinic pseudo-symmetry manifests itself by the presence of a pseudo *b*-glide operation along the *a*-axis of the triclinic cell, Fig. 3 ▸. Fig. 4 ▸ shows a least-squares overlay of one set of cations *A* and *B*, of the surrounding chloride anions and solvate mol­ecules and of a second cation. The pseudo-glide symmetry is mostly obeyed by the constituents of the asymmetric unit; the root-mean-square deviation for one overlaid pair of *A* and *B* cations is 0.138 Å. For the surrounding solvate mol­ecules, for the chloride anions and neighboring cations this is no longer the case. This can especially be seen for a second cation shown in Fig. 4 ▸ (on the left), which was not included in the calculation of the least-squares overlay fit, and shows easily recognizable positional shifts for its atoms related by pseudo-symmetry. The substantial deviation of the lattice from the ideal monoclinic symmetry (by 1.03 and 0.48° for α and γ, respectively) leads to an insufficient match and the increased deviations of atoms of the next and second next ions and solvate mol­ecules break the higher symmetry. The crystal under investigation did, however, show signs of slight twinning by pseudo-monoclinic symmetry. The application of a 180° rotation around reciprocal (2 0 1) (command ‘TWIN 1 0 0 0 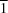 0 1 0 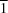
′ in *SHELXL*) resulted in a twinning ratio of 0.798 (3):0.202 (3), and *R*_1_ does increase by 2.6% if twinning is ignored during structure refinement.

**Figure 5:**
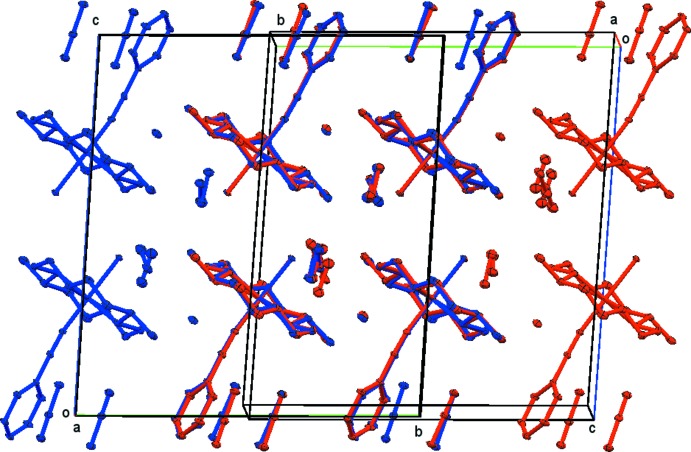
Overlays of a larger segment of the lattice of compound **1**. One of the overlaid cells was inverted for this purpose. The view is along the *a* axis of the original cell (blue atoms). The overlay is based on a least-squares fit of the four cobalt ions common to the overlaid structures.

Overlays of a larger segment of the lattice, along the *a* and *c*-axes, are shown in Figs. 5 ▸ and 6 ▸ (one of the overlaid cells was inverted for this purpose). The overlays are based on a least-squares fit of the four cobalt ions common to the overlaid structures.

**Figure 7:**
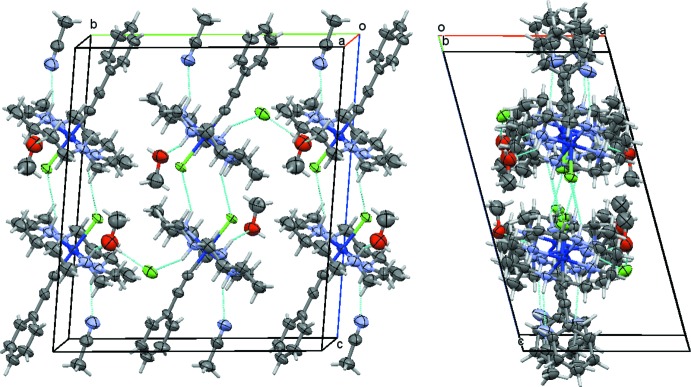
Hydrogen-bonding inter­actions in **1**, showing a segment of the ribbons formed by N—H⋯N, N—H⋯O, and N—H⋯Cl hydrogen bonds (symbolized by dashed turquois lines). Views are slightly tilted down the *a* axis (left) and the *b* axis (right). C—H⋯O inter­actions, omitted for clarity, connect parallel ribbons along the *a*-axis direction. Disorder of methanol mol­ecules is omitted for clarity.

The cations, anions, and solvate mol­ecules in each structure are connected through a series of inter­molecular hydrogen bonds (Figs. 7 ▸–10 ▸ ▸ ▸, see Tables 3 ▸ and 4 ▸ for metrical details and symmetry operators). In the chloride salt **1** of the mono­acetyl­ide, the ammonium N—H units of the macrocycle form N—H⋯N hydrogen bonds with the aceto­nitrile nitro­gen atom, N—H⋯O hydrogen bonds to the methanol oxygen, and N—H⋯Cl hydrogen bonds to both the inter­stitial chloride anions as well as the cobalt-bound chlorine. The chloride anions are also acceptors for O—H⋯Cl hydrogen bonds originating from the disordered methanol mol­ecules and for a series of weaker C—H⋯Cl hydrogen bonds from macrocyclic carbon atoms. The type and number of hydrogen bonds is essentially the same between the two halves of the structure related by pseudo-symmetry, but the exact metrics and numbers are slightly modulated. The N—H⋯N, N—H⋯O, and N—H⋯Cl hydrogen bonds, when combined, connect the cations, anions and solvate mol­ecules into ribbons that extend infinitely along the *b*-axis and are perpendicular to the *a*-axis, and exactly one unit cell thick in the *a*- and *c*-axis directions (Fig. 7 ▸). Perpendicular to the *a*-axis, the ribbons are terminated by methanol O atoms and chloride anions, which at their open sides are surrounded by hydrogen atoms from aliphatic C—H, CH_2_ and CH_3_ groups, thus connecting parallel ribbons with each other. Perpendicular to the *c*-axis, ribbons are lined by phenyl and methyl groups from the phenyl­acetyl­ide and the aceto­nitrile mol­ecules, respectively. Inter­actions with neighboring ribbons are van der Waals in nature.

**Figure 8:**
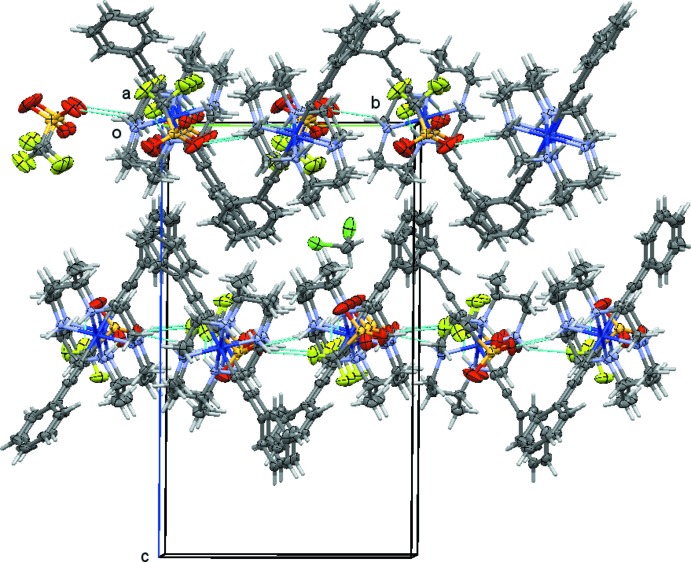
Hydrogen-bonding inter­actions in **2**, showing both layers connected by N—H⋯O, N—H⋯F hydrogen bonds. The top layer contains cations and anions of Co1 and S1, respectively, the bottom layer those of Co2 and S2. Hydrogen bonds are shown as dashed turquoise lines. Disorder of one of the tri­fluoro­methane­sulfonate anions and methyl­ene chloride is omitted for clarity. View is down the *a*-axis direction.

**Figure 9:**
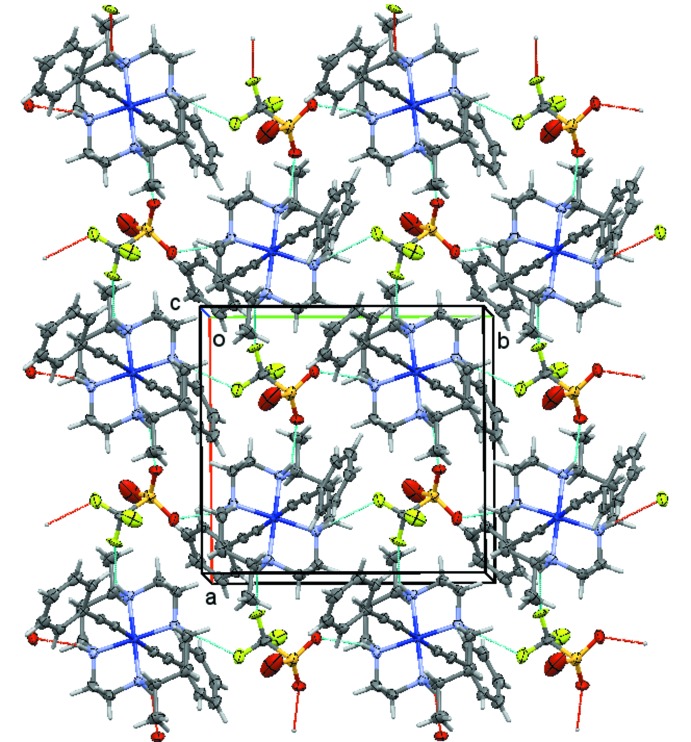
Hydrogen-bonding inter­actions in **2**, showing the hydrogen-bonded layer formed by cations and anions of Co1 and S1, respectively. Hydrogen bonds are depicted as dashed turquoise lines. View is slightly tilted down the *c* axis. Disorder of the tri­fluoro­methane­sulfonate anion is omitted for clarity.

**Figure 10:**
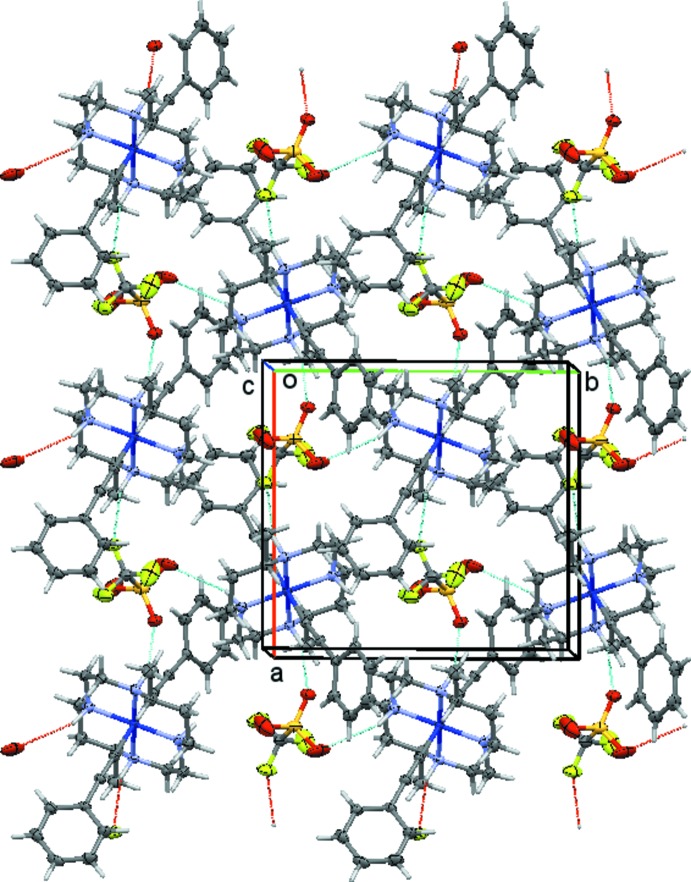
Hydrogen-bonding inter­actions in **2**, showing the hydrogen-bonded layer formed by cations and anions of Co2 and S2, respectively. Hydrogen bonds are depicted as dashed turquoise lines. View is slightly tilted down the *c* axis.

In the triflate salt **2** of the bis­acetyl­ide complex, the two cations form N—H⋯O and N—H⋯F hydrogen bonds with the two triflate anions (Fig. 8 ▸). The two mol­ecules have a distinctively different set of hydrogen bonds. The number of hydrogen bonds, their type (N—H⋯O *versus* N—H⋯F), and their strength varies substanti­ally between the ion pairs. The first of the two cations, involving nitro­gen atoms N1 through N4, features each two N—H⋯O and N—H⋯F hydrogen bonds (not counting duplicates from triflate disorder), Fig. 9 ▸. The second cation, involving nitro­gen atoms N5 through N8, makes three N—H⋯O hydrogen bonds, and one N—H⋯F (Fig. 10 ▸). On average, the hydrogen bonds involving this second mol­ecule are much weaker than those involving the first mol­ecule, with two of the N—H⋯O bonds and the N—H⋯F bond having donor–acceptor distances longer than 3.52 Å. For the first mol­ecule, only one exceeds a value of 3.5 Å, and this one is towards the minor moiety of the disordered triflate anion. The methyl­ene chloride halogen atoms do not act as acceptors for hydrogen bonds, but are involved in weak C—H⋯O hydrogen bonds towards one of the triflate anions.

## Synthesis and crystallization   

All reactions were carried out under ambient conditions. [Co^III^(DMC)Cl_2_]Cl was synthesized according to literature procedures (Hay *et al.*, 1984 ▸).
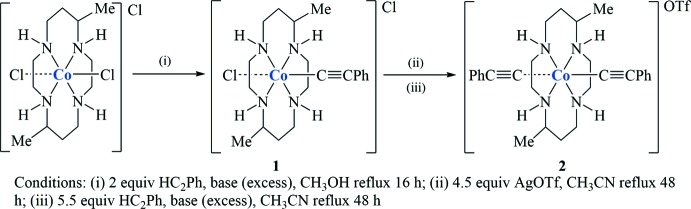


Preparation of [Co^III^(DMC)(C_2_Ph)Cl]Cl (**1**). [Co^III^(DMC)Cl_2_]Cl (200 mg, 0.51 mmol) was dissolved in 40 mL of methanol. Phenyl­acetyl­ene (0.12 mmol, 1.1 mmol) and Et_3_N (0.77 mL, 5.6 mmol) were added and the solution was refluxed overnight. Solvent was removed via rotary evaporation, and the solid was loaded onto a silica gel plug and eluted with CH_3_OH/EtOAc (*v*/*v*, 1:6) as a red fraction. The desired product was recrystallized from ether–methanol to afford 170 mg of a coral solid (73% based on [Co^III^(DMC)Cl_2_]Cl). Single crystals were grown from slow diffusion of ether into a methanol solution of **1**.

Data for [Co^III^(DMC)(C_2_Ph)Cl]Cl (**1**). ESI–MS: [*M*]^+^, 423.0. ^1^H NMR (300 MHz, CD_3_OD, δ): 7.55–7.41 (*m*, 2H), 7.37–7.25 (*m*, 2H), 7.25–7.15 (*m*, 1H), 5.36 (*s*, 2H), 4.23 (*s*, 2H), 3.21–2.46 (*m*, 14H), 1.93–1.84 (*m*, 2H), 1.53–1.48 (*m*, 2H), 1.30 (*dd*, *J* = 6.9, 4.7 Hz, 6H). Visible spectra, λ_max_ [nm, ∊ (*M* ^−1^, cm^−1^)]: 256 (36, 800), 493 (101); IR (cm^−1^): C≡C: 2122 (*m*).

Preparation of [Co^III^(DMC)(C_2_Ph)_2_]OTf (**2**). Compound **1** (150 mg, 0.33 mmol) and AgOTf (384 mg, 1.49 mmol) were dissolved in 50 mL of CH_3_CN and refluxed for 48 h. The precipitate that formed was filtered out, and 3.1 mL (22 mmol) of Et_3_N and 0.20 mL (1.8 mmol) of phenyl­acetyl­ene were added and the solution was refluxed for 48 h. The solution was purified over a silica gel plug and the product eluted with CH_3_OH/EtOAc (*v*/*v*, 1:8). A pale-yellow fraction was collected and recrystallized from ether–methanol to afford 102 mg of a yellow solid (47% based on **1**). Single crystals were grown from slow diffusion of *n*-hexa­nes into a CH_3_OH/CH_2_Cl­_2_ (*v*/*v*, 1:9) solution of **2**.

Data for [Co^III^(DMC)(C_2_Ph)_2_]OTf (**2**)*.* ESI–MS: [*M*]^+^, 489.0. ^1^H NMR (300 MHz, CD_3_OD, δ): 7.58–7.42 (*m*, 4H), 7.36–7.24 (*m*, 4H), 7.22–7.13 (*m*, 2H), 4.90 (*s*, 2H), 3.84 (*s*, 2H), 3.30–3.01 (*m*, 6H), 2.81–2.78 (*m*, 2H), 2.68–2.63 (*m*, 3H), 2.50–2.43 (*m*, 3H), 1.83 (*d*, 2H), 1.38 (*d*, 2H), 1.27 (*d*, 6H). Visible spectra, λ_max_ [nm, ∊ (*M* ^−1^, cm^−1^)]: 271 (40, 800), 464 (64.5); IR (cm^−1^): C≡C: 2102 (*m*).

## Refinement   

Crystal data, data collection and structure refinement details are summarized in Table 5 ▸. H atoms attached to carbon and nitro­gen atoms and hydroxyl hydrogen atoms were positioned geometrically and constrained to ride on their parent atoms. Carbon-to-hydrogen bond distances were constrained to 0.95 Å for aromatic C—H. Aliphatic C—H, CH_2_, and CH_3_ moieties were constrained to 1.00, 0.99 and 0.98 Å, respect­ively. N—H distances were constrained to 0.88 Å and O—H distances to 0.84 Å. Methyl and hydroxyl H atoms were allowed to rotate, but not to tip, to best fit the experimental electron density. *U*_iso_(H) values were set to a multiple of *U*_eq_(C) with 1.5 for OH and CH_3_, and 1.2 for N—H and C—H units, respectively.

The structure of compound **1** exhibits pseudo-symmetry and emulates a double-volume *C*-centered monoclinic cell in space group *C*2/*c*. The pseudo-symmetry is only approximate, and the α and γ angles deviate substanti­ally from the expected 90° for monoclinic (approximate cell dimensions: 34.71, 9.69, 15.67, 88.97, 93.41, 89.52). The structure is, however, twinned by a symmetry operator of the approximate larger monoclinic cell, by a 180° rotation around the [201] direction in reciprocal space (of the actual triclinic cell). Application of the twin matrix 1 0 0, 0 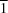 0, 1 0 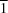 yielded a twin ratio of 0.798 (3):0.202 (3).

In the structure of compound **1**, each methanol group was refined with two-component disorder with a shared occupancy ratio for the two sites. The C—O bond lengths were restrained to 1.427 (20) Å. Each minor occupancy component was restrained to be similar the respective major occupancy component (SAME command of *SHELXL*, s.u. = 0.02 Å). The *U* ^ij^ components for atoms within 2.0 Å were restrained to be similar (SIMU command of *SHELXL*, s.u. = 0.01 Å^2^). The alcohol hydrogen atom to neighboring chloride distances were restrained based on hydrogen-bonding considerations. Subject to these conditions, the occupancy rates refined to 0.643 (16) and 0.357 (16).

In the structure of compound **2**, the S1 triflate anion was refined with two-component disorder. Each moiety was restrained to have a similar geometry as the S2 triflate anion (SAME command of *SHELXL*, s.u. = 0.02 Å). The *U* ^ij^ components for disordered atoms within 2.0 Å were restrained to be similar (SIMU command of *SHELXL*, s.u. = 0.01 Å^2^). Subject to these conditions, the occupancy factors refined to 0.503 (22) and 0.497 (22). The di­chloro­methane mol­ecule was refined with two-component disorder. The minor occupancy component was restrained to have a similar geometry as the major occupancy component (SIMU command of *SHELXL*, s.u. = 0.01 Å^2^). The *U* ^ij^ components for atoms within 2.0 Å were restrained to be similar (SIMU command of *SHELXL*, s.u. = 0.01 Å^2^). Subject to these conditions, the occupancy factors refined to 0.545 (12) and 0.455 (12).

## Supplementary Material

Crystal structure: contains datablock(s) 1, 2, global. DOI: 10.1107/S2056989018003997/vm2209sup1.cif

Structure factors: contains datablock(s) 1. DOI: 10.1107/S2056989018003997/vm22091sup2.hkl

Structure factors: contains datablock(s) 2. DOI: 10.1107/S2056989018003997/vm22092sup3.hkl

CCDC references: 1828222, 1828221

Additional supporting information:  crystallographic information; 3D view; checkCIF report

## Figures and Tables

**Figure 6:**
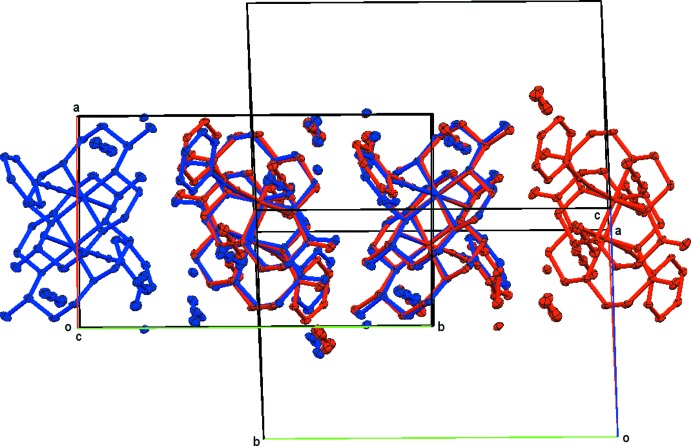
Overlays of a larger segment of the lattice of compound **1**. One of the overlaid cells was inverted for this purpose. The view is along the *c* axis of the original cell (blue atoms). The overlay is based on a least-squares fit of the four cobalt ions common to the overlaid structures.

**Table 1:** Selected geometric parameters (Å, °) for **1** [Chem scheme1]

Co1*A*—C1*A*	1.893 (7)	Co2*B*—C1*B*	1.905 (7)
Co1*A*—N3*A*	1.968 (7)	Co2*B*—N1*B*	1.960 (6)
Co1*A*—N1*A*	1.973 (7)	Co2*B*—N3*B*	1.960 (7)
Co1*A*—N2*A*	1.979 (6)	Co2*B*—N2*B*	1.996 (7)
Co1*A*—N4*A*	1.982 (7)	Co2*B*—N4*B*	1.999 (7)
Co1*A*—Cl1*A*	2.3270 (18)	Co2*B*—Cl1*B*	2.3233 (18)
C1*A*—C2*A*	1.189 (10)	C1*B*—C2*B*	1.168 (9)
C2*A*—C3*A*	1.444 (10)	C2*B*—C3*B*	1.437 (9)
			
C1*A*—Co1*A*—N3*A*	89.8 (3)	C1*B*—Co2*B*—N1*B*	89.9 (2)
C1*A*—Co1*A*—N1*A*	89.6 (3)	C1*B*—Co2*B*—N3*B*	89.3 (3)
N3*A*—Co1*A*—N1*A*	179.1 (3)	N1*B*—Co2*B*—N3*B*	179.3 (3)
C1*A*—Co1*A*—N2*A*	91.7 (3)	C1*B*—Co2*B*—N2*B*	92.2 (3)
N3*A*—Co1*A*—N2*A*	94.4 (3)	N1*B*—Co2*B*—N2*B*	86.7 (3)
N1*A*—Co1*A*—N2*A*	86.2 (3)	N3*B*—Co2*B*—N2*B*	93.4 (3)
C1*A*—Co1*A*—N4*A*	88.2 (3)	C1*B*—Co2*B*—N4*B*	88.0 (3)
N3*A*—Co1*A*—N4*A*	85.6 (3)	N1*B*—Co2*B*—N4*B*	92.9 (3)
N1*A*—Co1*A*—N4*A*	93.9 (3)	N3*B*—Co2*B*—N4*B*	87.1 (3)
N2*A*—Co1*A*—N4*A*	179.8 (3)	N2*B*—Co2*B*—N4*B*	179.5 (3)
C1*A*—Co1*A*—Cl1*A*	177.7 (2)	C1*B*—Co2*B*—Cl1*B*	178.0 (2)
N3*A*—Co1*A*—Cl1*A*	88.0 (2)	N1*B*—Co2*B*—Cl1*B*	92.08 (17)
N1*A*—Co1*A*—Cl1*A*	92.66 (18)	N3*B*—Co2*B*—Cl1*B*	88.6 (2)
N2*A*—Co1*A*—Cl1*A*	88.08 (18)	N2*B*—Co2*B*—Cl1*B*	87.81 (18)
N4*A*—Co1*A*—Cl1*A*	92.09 (18)	N4*B*—Co2*B*—Cl1*B*	92.00 (17)
C2*A*—C1*A*—Co1*A*	171.3 (7)	C2*B*—C1*B*—Co2*B*	171.8 (6)

**Table 2:** Selected geometric parameters (Å, °) for **2** [Chem scheme1]

Co1—C9	1.926 (2)	Co2—C37	1.9262 (19)
Co1—C1	1.927 (2)	Co2—C29	1.9273 (19)
Co1—N1	1.9768 (19)	Co2—N7	1.9789 (18)
Co1—N3	1.982 (2)	Co2—N5	1.9835 (18)
Co1—N4	1.9985 (18)	Co2—N6	2.0067 (17)
Co1—N2	2.0126 (18)	Co2—N8	2.0071 (16)
C1—C2	1.215 (3)	C29—C30	1.214 (3)
C2—C3	1.438 (3)	C30—C31	1.441 (3)
C9—C10	1.206 (3)	C37—C38	1.212 (3)
C10—C11	1.435 (3)	C38—C39	1.440 (3)
			
C9—Co1—C1	179.67 (9)	C37—Co2—C29	177.67 (9)
C9—Co1—N1	87.08 (9)	C37—Co2—N7	92.41 (8)
C1—Co1—N1	92.91 (8)	C29—Co2—N7	88.10 (8)
C9—Co1—N3	91.84 (9)	C37—Co2—N5	87.84 (8)
C1—Co1—N3	88.17 (9)	C29—Co2—N5	91.66 (8)
N1—Co1—N3	178.87 (8)	N7—Co2—N5	179.53 (8)
C9—Co1—N4	89.79 (8)	C37—Co2—N6	90.34 (8)
C1—Co1—N4	90.54 (8)	C29—Co2—N6	87.36 (8)
N1—Co1—N4	93.92 (8)	N7—Co2—N6	94.17 (8)
N3—Co1—N4	86.43 (8)	N5—Co2—N6	86.23 (8)
C9—Co1—N2	90.20 (8)	C37—Co2—N8	89.93 (8)
C1—Co1—N2	89.47 (8)	C29—Co2—N8	92.37 (8)
N1—Co1—N2	86.35 (8)	N7—Co2—N8	86.38 (7)
N3—Co1—N2	93.30 (8)	N5—Co2—N8	93.23 (8)
N4—Co1—N2	179.73 (9)	N6—Co2—N8	179.38 (8)
C2—C1—Co1	174.06 (19)	C30—C29—Co2	171.40 (19)

**Table 3:** Hydrogen-bond geometry (Å, °) for **1** [Chem scheme1]

*D*—H⋯*A*	*D*—H	H⋯*A*	*D*⋯*A*	*D*—H⋯*A*
N1*A*—H1*NA*⋯Cl2*B* ^i^	1.00	2.40	3.265 (6)	144
N2*A*—H2*NA*⋯Cl1*A* ^ii^	1.00	2.91	3.684 (7)	134
N3*A*—H3*N*⋯O1*A*	1.00	2.02	2.844 (16)	138
N3*A*—H3*N*⋯O1*C*	1.00	2.14	3.10 (3)	160
N4*A*—H4*N*⋯N5*A*	1.00	2.30	3.185 (10)	147
N1*B*—H1*BN*⋯Cl2*A*	1.00	2.40	3.251 (6)	143
N2*B*—H2*BN*⋯Cl1*B* ^iii^	1.00	2.83	3.607 (6)	135
N3*B*—H3*BN*⋯O1*B*	1.00	2.06	2.863 (13)	136
N3*B*—H3*BN*⋯O1*D*	1.00	2.08	3.03 (3)	158
N4*B*—H4*BN*⋯N5*B*	1.00	2.26	3.148 (10)	147
O1*A*—H1*OA*⋯Cl2*A*	0.84	2.09	2.899 (18)	163
O1*B*—H1*OB*⋯Cl2*B*	0.84	2.19	2.979 (13)	157
O1*C*—H1*OC*⋯Cl2*A*	0.84	2.40	3.16 (4)	152
O1*D*—H1*OD*⋯Cl2*B*	0.84	2.30	3.13 (3)	168
C9*A*—H9*A*⋯Cl1*A* ^ii^	0.99	2.92	3.631 (9)	130
C10*A*—H10*A*⋯Cl1*A* ^ii^	0.99	2.96	3.531 (8)	118
C14*A*—H14*B*⋯Cl2*A*	0.99	2.98	3.884 (10)	152
C16*A*—H16*A*⋯Cl1*A*	1.00	2.81	3.373 (9)	116
C17*A*—H17*B*⋯O1*A* ^iv^	0.99	2.46	3.44 (2)	169
C18*A*—H18*A*⋯Cl1*A*	0.99	2.78	3.339 (9)	116
C20*A*—H20*B*⋯Cl2*A* ^iv^	0.98	2.88	3.834 (11)	164
C22*A*—H22*A*⋯Cl2*B* ^v^	0.98	2.82	3.669 (11)	146
C9*B*—H9*C*⋯Cl2*A*	0.99	2.97	3.498 (8)	114
C10*B*—H10*C*⋯Cl1*B* ^iii^	0.99	2.89	3.485 (7)	120
C16*B*—H16*B*⋯Cl1*B*	1.00	2.85	3.407 (9)	116
C17*B*—H17*C*⋯O1*B* ^vi^	0.99	2.63	3.479 (18)	144
C18*B*—H18*C*⋯Cl1*B*	0.99	2.83	3.358 (9)	114
C22*B*—H22*E*⋯Cl2*A* ^vii^	0.98	2.81	3.583 (12)	137

Symmetry codes: (i) 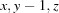; (ii) 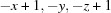; (iii) 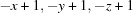; (iv) 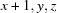; (v) 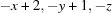; (vi) 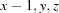; (vii) 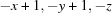.

**Table 4:** Hydrogen-bond geometry (Å, °) for **2** [Chem scheme1]

*D*—H⋯*A*	*D*—H	H⋯*A*	*D*⋯*A*	*D*—H⋯*A*
N1—H1*N*⋯F2^i^	1.00	2.43	3.298 (9)	145
N1—H1*N*⋯F2*B* ^i^	1.00	2.59	3.504 (14)	151
N2—H2*N*⋯F1^ii^	1.00	2.61	3.482 (13)	146
N2—H2*N*⋯F1*B* ^ii^	1.00	2.61	3.491 (11)	148
N3—H3*N*⋯O2^iii^	1.00	2.06	2.947 (15)	147
N3—H3*N*⋯O2*B* ^iii^	1.00	2.13	3.053 (18)	153
N4—H4*N*⋯O3	1.00	2.14	3.001 (11)	143
N4—H4*N*⋯O3*B*	1.00	2.31	3.183 (12)	145
C21—H21*B*⋯Cl1*B*	0.99	2.94	3.779 (9)	144
C22—H22*B*⋯O3^iii^	0.99	2.49	3.401 (14)	152
C23—H23*B*⋯F2	0.99	2.59	3.419 (11)	142
C23—H23*B*⋯F2*B*	0.99	2.64	3.543 (12)	152
N5—H5*N*⋯O4^iv^	1.00	2.92	3.523 (4)	119
N6—H6*N*⋯F5	1.00	2.29	3.211 (2)	153
N7—H7*N*⋯O6^v^	1.00	2.69	3.575 (4)	148
N8—H8*N*⋯O5^ii^	1.00	2.05	2.960 (2)	150
C46—H46*B*⋯O6	0.99	2.52	3.483 (3)	163
C49—H49*B*⋯O5^v^	0.99	2.57	3.408 (3)	142
C51—H51*A*⋯F4^ii^	0.99	2.62	3.590 (3)	167
C52—H52⋯Cl1*B*	1.00	2.86	3.637 (8)	136
C54—H54*A*⋯O4^iv^	0.99	2.59	3.307 (4)	129
C59—H59*B*⋯O1	0.99	2.24	3.169 (13)	155
C59*B*—H59*C*⋯O1*B*	0.99	2.39	3.027 (12)	122

Symmetry codes: (i) 
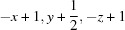
; (ii) 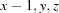; (iii) 
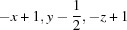
; (iv) 
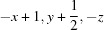
; (v) 
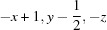
.

**Table 5:** Experimental details

	**1**	**2**
Crystal data		
Chemical formula	[Co(C_8_H_5_)Cl(C_12_H_28_N_4_)]Cl·C_2_H_3_N·CH_4_O	[Co(C_8_H_5_)_2_(C_12_H_28_N_4_)]_2_(CF_3_SO_3_)_2_·CH_2_Cl_2_
*M*_r_	532.43	1362.17
Crystal system, space group	Triclinic, *P* 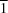	Monoclinic, *P*2_1_
Temperature (K)	150	150
*a*, *b*, *c* (Å)	9.6903 (13), 15.668 (2), 17.985 (2)	12.0263 (7), 12.3999 (5), 21.9164 (14)
α, β, γ (°)	86.430 (5), 74.848 (4), 88.970 (5)	90, 105.3260 (14), 90
*V* (Å^3^)	2630.6 (6)	3152.1 (3)
*Z*	4	2
Radiation type	Mo *K*α	Mo *K*α
μ (mm^−1^)	0.88	0.75
Crystal size (mm)	0.36 × 0.25 × 0.09	0.40 × 0.30 × 0.10

Data collection		
Diffractometer	Bruker AXS D8 Quest CMOS	Bruker AXS D8 Quest CMOS
Absorption correction	Multi-scan (*SADABS*; Krause et al., 2015 ▸)	Multi-scan (*SADABS*; Krause et al., 2015 ▸)
*T*_min_, *T*_max_	0.190, 0.263	0.660, 0.747
No. of measured, independent and observed [*I* > 2σ(*I*)] reflections	44192, 9687, 7643	54456, 22462, 18066
*R*_int_	0.095	0.026
(sin θ/λ)_max_ (Å^−1^)	0.610	0.771

Refinement		
*R*[*F* ^2^ > 2σ(*F* ^2^)], *wR*(*F* ^2^), *S*	0.087, 0.241, 1.10	0.034, 0.078, 1.01
No. of reflections	9687	22462
No. of parameters	628	872
No. of restraints	79	349
H-atom treatment	H-atom parameters constrained	H-atom parameters constrained
Δρ_max_, Δρ_min_ (e Å^−3^)	0.93, −1.41	0.44, −0.56
Absolute structure	–	Flack *x* determined using 6987 quotients [(*I* ^+^)−(*I* ^−^)]/[(*I* ^+^)+(*I* ^−^)] (Parsons et al., 2013 ▸)
Absolute structure parameter	–	−0.003 (3)

Computer programs: *APEX3* and *SAINT* (Bruker, 2016 ▸), *SHELXS97* (Sheldrick, 2008 ▸), *SHELXL2017* and *SHELXL2018* (Sheldrick, 2015 ▸), *SHELXLE* (Hübschle *et al.*, 2011 ▸), *Mercury* (Macrae *et al.*, 2008 ▸) and *publCIF* (Westrip, 2010 ▸).

## supplementary crystallographic information

### Chlorido(5,12-dimethyl-1,4,8,11-tetraazacyclotetradecane)(phenylethynyl)cobalt(III) chloride–acetonitrile–methanol (1/1/1) (1) Crystal data

**Table:**

[Co(C_8_H_5_)Cl(C_12_H_28_N_4_)]Cl·C_2_H_3_N·CH_4_O	*Z* = 4
*M**_r_* = 532.43	*F*(000) = 1128
Triclinic, *P*1	*D*_x_ = 1.344 Mg m^−^^3^
*a* = 9.6903 (13) Å	Mo *K*α radiation, λ = 0.71073 Å
*b* = 15.668 (2) Å	Cell parameters from 9887 reflections
*c* = 17.985 (2) Å	θ = 3.1–28.4°
α = 86.430 (5)°	µ = 0.88 mm^−^^1^
β = 74.848 (4)°	*T* = 150 K
γ = 88.970 (5)°	Plate, orange
*V* = 2630.6 (6) Å^3^	0.36 × 0.25 × 0.09 mm

### Chlorido(5,12-dimethyl-1,4,8,11-tetraazacyclotetradecane)(phenylethynyl)cobalt(III) chloride–acetonitrile–methanol (1/1/1) (1) Data collection

**Table:**

Bruker AXS D8 Quest CMOS diffractometer	9687 independent reflections
Radiation source: sealed tube X-ray source	7643 reflections with *I* > 2σ(*I*)
Triumph curved graphite crystal monochromator	*R*_int_ = 0.095
ω and phi scans	θ_max_ = 25.7°, θ_min_ = 3.0°
Absorption correction: multi-scan (SADABS; Krause et al., 2015)	*h* = −11→11
*T*_min_ = 0.190, *T*_max_ = 0.263	*k* = −19→19
44192 measured reflections	*l* = −21→21

### Chlorido(5,12-dimethyl-1,4,8,11-tetraazacyclotetradecane)(phenylethynyl)cobalt(III) chloride–acetonitrile–methanol (1/1/1) (1) Refinement

**Table:**

Refinement on *F*^2^	Primary atom site location: structure-invariant direct methods
Least-squares matrix: full	Secondary atom site location: difference Fourier map
*R*[*F*^2^ > 2σ(*F*^2^)] = 0.087	Hydrogen site location: mixed
*wR*(*F*^2^) = 0.241	H-atom parameters constrained
*S* = 1.10	*w* = 1/[σ^2^(*F*_o_^2^) + (0.0698*P*)^2^ + 11.8383*P*] where *P* = (*F*_o_^2^ + 2*F*_c_^2^)/3
9687 reflections	(Δ/σ)_max_ < 0.001
628 parameters	Δρ_max_ = 0.93 e Å^−^^3^
79 restraints	Δρ_min_ = −1.40 e Å^−^^3^

### Chlorido(5,12-dimethyl-1,4,8,11-tetraazacyclotetradecane)(phenylethynyl)cobalt(III) chloride–acetonitrile–methanol (1/1/1) (1) Special details

**Table:**

Geometry. All esds (except the esd in the dihedral angle between two l.s. planes) are estimated using the full covariance matrix. The cell esds are taken into account individually in the estimation of esds in distances, angles and torsion angles; correlations between esds in cell parameters are only used when they are defined by crystal symmetry. An approximate (isotropic) treatment of cell esds is used for estimating esds involving l.s. planes.
Refinement. Refined as a 2-component twin. The structure exhibits pseudo-symmetry and emulates a double the volume C-centered monoclinic cell in space group C2/c. The pseudosymmetry is only approximate, and angles deviate substantially from the expected 90 degrees for monoclinic (approximate cell dimensions: 34.71, 9.69, 15.67, 88.97, 93.41, 89.52). The structure is however twinned by a symmetry operator of the approximate larger monoclinic cell, by a 180 degree rotation around the 2 0 1 direction in reciprocal space (of the actual triclinic cell). Application of the twin matrix 1 0 0, 0 -1 0, 1 0 -1 yielded a twin ratio of 0.798 (3) to 0.202 (3). Each methanol moiety was refined with two component disorder, with a shared occupancy ratio for the two sites. The C-O bond lengths were restrained to 1.427 (20) Angstrom. Alcohol hydrogen atom to neighboring chloride distances were restrained based on hydrogen bonding considerations. Subject to these conditions, the occupancy rates refined to 0.643 (16) and 0.357 (16).

### Chlorido(5,12-dimethyl-1,4,8,11-tetraazacyclotetradecane)(phenylethynyl)cobalt(III) chloride–acetonitrile–methanol (1/1/1) (1) Fractional atomic coordinates and isotropic or equivalent isotropic displacement parameters (Å^2^)

**Table:**

	*x*	*y*	*z*	*U*_iso_*/*U*_eq_	Occ. (<1)
Co1A	0.61768 (10)	0.01471 (6)	0.30868 (5)	0.0416 (3)	
N1A	0.7788 (7)	−0.0419 (4)	0.3401 (4)	0.0540 (16)	
H1NA	0.829233	−0.077073	0.296580	0.065*	
N2A	0.4963 (7)	−0.0762 (4)	0.3738 (4)	0.0499 (15)	
H2NA	0.450208	−0.050764	0.423916	0.060*	
N3A	0.4587 (7)	0.0713 (5)	0.2757 (4)	0.0587 (17)	
H3N	0.406483	0.106342	0.318867	0.070*	
N4A	0.7391 (7)	0.1056 (4)	0.2432 (4)	0.0503 (15)	
H4N	0.785258	0.079950	0.193153	0.060*	
Cl1A	0.5633 (2)	0.09833 (12)	0.41506 (10)	0.0511 (5)	
Cl2A	0.2600 (3)	0.32533 (17)	0.25087 (14)	0.0737 (7)	
C1A	0.6550 (7)	−0.0519 (4)	0.2213 (4)	0.0400 (14)	
C2A	0.6751 (8)	−0.0840 (5)	0.1612 (4)	0.0483 (17)	
C3A	0.6889 (8)	−0.1197 (4)	0.0875 (4)	0.0464 (16)	
C4A	0.8031 (8)	−0.1724 (5)	0.0545 (4)	0.0500 (18)	
H4A	0.874578	−0.186079	0.080791	0.060*	
C5A	0.8123 (10)	−0.2047 (6)	−0.0159 (5)	0.061 (2)	
H5A	0.891119	−0.240020	−0.038270	0.074*	
C6A	0.7076 (10)	−0.1865 (6)	−0.0550 (5)	0.063 (2)	
H6A	0.713826	−0.209682	−0.103368	0.075*	
C7A	0.5958 (10)	−0.1346 (7)	−0.0227 (5)	0.068 (2)	
H7A	0.524563	−0.120991	−0.049218	0.082*	
C8A	0.5855 (9)	−0.1021 (6)	0.0475 (5)	0.062 (2)	
H8A	0.506234	−0.066898	0.069388	0.075*	
C9A	0.7189 (10)	−0.1029 (6)	0.4069 (5)	0.063 (2)	
H9A	0.688548	−0.072062	0.454890	0.076*	
H9B	0.792020	−0.145826	0.412962	0.076*	
C10A	0.5943 (10)	−0.1457 (5)	0.3917 (5)	0.060 (2)	
H10A	0.544055	−0.181895	0.437502	0.072*	
H10B	0.626109	−0.182348	0.347441	0.072*	
C11A	0.3771 (10)	−0.1130 (6)	0.3467 (5)	0.068 (2)	
H11A	0.420573	−0.144014	0.299114	0.082*	
C12A	0.2813 (10)	−0.0412 (8)	0.3254 (7)	0.084 (3)	
H12A	0.196174	−0.067506	0.315582	0.101*	
H12B	0.247585	−0.005835	0.370450	0.101*	
C13A	0.3507 (9)	0.0165 (7)	0.2566 (6)	0.073 (3)	
H13A	0.397348	−0.018234	0.212805	0.088*	
H13B	0.277209	0.052945	0.241060	0.088*	
C14A	0.5195 (10)	0.1320 (6)	0.2085 (5)	0.065 (2)	
H14A	0.551017	0.100970	0.160665	0.078*	
H14B	0.446823	0.174949	0.201806	0.078*	
C15A	0.6434 (11)	0.1746 (5)	0.2251 (5)	0.063 (2)	
H15A	0.694739	0.211069	0.179653	0.075*	
H15B	0.610502	0.210975	0.269437	0.075*	
C16A	0.8591 (10)	0.1420 (6)	0.2708 (5)	0.066 (2)	
H16A	0.815270	0.171724	0.319035	0.080*	
C17A	0.9542 (9)	0.0703 (7)	0.2910 (6)	0.077 (3)	
H17A	0.987990	0.035929	0.245309	0.093*	
H17B	1.039348	0.096507	0.300940	0.093*	
C18A	0.8889 (9)	0.0126 (7)	0.3570 (6)	0.071 (3)	
H18A	0.844549	0.046511	0.401819	0.085*	
H18B	0.963817	−0.024104	0.370752	0.085*	
C19A	0.2892 (13)	−0.1762 (8)	0.4063 (6)	0.093 (4)	
H19A	0.210307	−0.197073	0.387651	0.140*	
H19B	0.250407	−0.148039	0.454590	0.140*	
H19C	0.349609	−0.224409	0.415455	0.140*	
C20A	0.9448 (12)	0.2077 (8)	0.2120 (6)	0.095 (4)	
H20A	0.986244	0.180671	0.163398	0.142*	
H20B	1.021608	0.229986	0.231493	0.142*	
H20C	0.881893	0.254689	0.203115	0.142*	
N5A	0.8348 (11)	0.0938 (6)	0.0607 (5)	0.084 (3)	
C21A	0.8507 (10)	0.0751 (6)	−0.0005 (5)	0.065 (2)	
C22A	0.8738 (13)	0.0504 (7)	−0.0794 (5)	0.082 (3)	
H22A	0.936081	0.092343	−0.114642	0.098*	
H22B	0.781824	0.048281	−0.092333	0.098*	
H22C	0.919151	−0.006138	−0.084429	0.098*	
O1A	0.2181 (17)	0.1695 (11)	0.3497 (10)	0.101 (4)	0.643 (16)
H1OA	0.212874	0.212254	0.319941	0.152*	0.643 (16)
C23A	0.180 (3)	0.1931 (17)	0.4250 (12)	0.112 (6)	0.643 (16)
H23A	0.109879	0.239644	0.430125	0.168*	0.643 (16)
H23B	0.265050	0.212429	0.438940	0.168*	0.643 (16)
H23C	0.138126	0.143948	0.459322	0.168*	0.643 (16)
O1C	0.242 (3)	0.1838 (19)	0.386 (2)	0.096 (6)	0.357 (16)
H1OC	0.239863	0.232379	0.364083	0.144*	0.357 (16)
C23C	0.104 (4)	0.163 (3)	0.434 (3)	0.113 (7)	0.357 (16)
H23G	0.086964	0.193084	0.481681	0.170*	0.357 (16)
H23H	0.098976	0.100852	0.446920	0.170*	0.357 (16)
H23I	0.032010	0.179346	0.406757	0.170*	0.357 (16)
Co2B	0.59189 (10)	0.51435 (6)	0.30908 (5)	0.0417 (3)	
N1B	0.3958 (6)	0.4714 (4)	0.3312 (3)	0.0473 (15)	
H1BN	0.393494	0.435702	0.287316	0.057*	
N2B	0.6267 (7)	0.4190 (4)	0.3809 (3)	0.0475 (14)	
H2BN	0.617373	0.444572	0.431508	0.057*	
N3B	0.7887 (7)	0.5564 (4)	0.2859 (4)	0.0551 (16)	
H3BN	0.792992	0.591556	0.329769	0.066*	
N4B	0.5553 (7)	0.6099 (4)	0.2376 (3)	0.0480 (15)	
H4BN	0.562605	0.583899	0.187359	0.058*	
Cl1B	0.5340 (2)	0.59978 (12)	0.41394 (10)	0.0532 (5)	
Cl2B	1.0093 (2)	0.81517 (16)	0.25885 (14)	0.0677 (6)	
C1B	0.6461 (7)	0.4456 (4)	0.2224 (4)	0.0383 (14)	
C2B	0.6812 (8)	0.4126 (5)	0.1638 (4)	0.0452 (16)	
C3B	0.7320 (8)	0.3759 (4)	0.0903 (4)	0.0453 (16)	
C4B	0.6445 (9)	0.3266 (5)	0.0600 (4)	0.0540 (19)	
H4B	0.547887	0.316858	0.088000	0.065*	
C5B	0.6974 (11)	0.2913 (6)	−0.0111 (5)	0.066 (2)	
H5B	0.637176	0.256167	−0.030271	0.079*	
C6B	0.8343 (10)	0.3064 (6)	−0.0535 (5)	0.064 (2)	
H6B	0.867752	0.284395	−0.103101	0.077*	
C7B	0.9244 (10)	0.3541 (7)	−0.0238 (5)	0.068 (2)	
H7B	1.020872	0.363277	−0.052210	0.081*	
C8B	0.8729 (9)	0.3884 (6)	0.0479 (5)	0.057 (2)	
H8B	0.935195	0.420783	0.068086	0.069*	
C9B	0.3724 (9)	0.4117 (5)	0.4000 (4)	0.054 (2)	
H9C	0.289548	0.374362	0.402959	0.065*	
H9D	0.352400	0.443928	0.447242	0.065*	
C10B	0.5058 (9)	0.3582 (5)	0.3935 (4)	0.053 (2)	
H10C	0.498040	0.321866	0.441468	0.063*	
H10D	0.520186	0.320845	0.349743	0.063*	
C11B	0.7685 (10)	0.3717 (6)	0.3631 (5)	0.059 (2)	
H11B	0.777874	0.340922	0.314997	0.071*	
C12B	0.8899 (10)	0.4348 (7)	0.3487 (6)	0.071 (2)	
H12C	0.874830	0.470030	0.394028	0.085*	
H12D	0.980056	0.402558	0.344698	0.085*	
C13B	0.9071 (9)	0.4926 (6)	0.2788 (6)	0.069 (2)	
H13C	0.910668	0.458133	0.234084	0.083*	
H13D	0.999091	0.523341	0.268845	0.083*	
C14B	0.8114 (9)	0.6163 (6)	0.2177 (5)	0.060 (2)	
H14C	0.832464	0.584310	0.170222	0.072*	
H14D	0.893730	0.653966	0.215046	0.072*	
C15B	0.6792 (10)	0.6690 (5)	0.2234 (5)	0.058 (2)	
H15C	0.665991	0.707561	0.266245	0.069*	
H15D	0.687243	0.704296	0.174867	0.069*	
C16B	0.4142 (9)	0.6575 (6)	0.2560 (5)	0.058 (2)	
H16B	0.403605	0.685615	0.305583	0.070*	
C17B	0.2959 (10)	0.5940 (6)	0.2674 (6)	0.066 (2)	
H17C	0.205170	0.625546	0.271000	0.080*	
H17D	0.313897	0.560080	0.221097	0.080*	
C18B	0.2771 (8)	0.5339 (6)	0.3366 (5)	0.064 (2)	
H18C	0.269110	0.567323	0.382512	0.077*	
H18D	0.186457	0.502234	0.344353	0.077*	
C19B	0.7802 (12)	0.3064 (6)	0.4274 (6)	0.071 (2)	
H19D	0.772775	0.335568	0.474926	0.107*	
H19E	0.872543	0.276960	0.412692	0.107*	
H19F	0.702753	0.264613	0.435934	0.107*	
C20B	0.4069 (12)	0.7268 (6)	0.1935 (5)	0.075 (3)	
H20D	0.428670	0.701604	0.143168	0.113*	
H20E	0.476638	0.771659	0.192445	0.113*	
H20F	0.310578	0.751593	0.204690	0.113*	
N5B	0.6455 (13)	0.5963 (6)	0.0575 (5)	0.094 (3)	
C21B	0.6682 (12)	0.5719 (6)	−0.0016 (5)	0.067 (2)	
C22B	0.6983 (15)	0.5401 (8)	−0.0779 (6)	0.091 (4)	
H22D	0.749261	0.485480	−0.078549	0.109*	
H22E	0.757615	0.581550	−0.115273	0.109*	
H22F	0.608273	0.531768	−0.091733	0.109*	
O1B	0.9508 (15)	0.6559 (9)	0.3612 (7)	0.080 (3)	0.643 (16)
H1OB	0.941678	0.702341	0.337067	0.120*	0.643 (16)
C23B	0.927 (3)	0.669 (2)	0.4407 (11)	0.097 (5)	0.643 (16)
H23D	0.824619	0.680101	0.463023	0.145*	0.643 (16)
H23E	0.981943	0.719040	0.446957	0.145*	0.643 (16)
H23F	0.956472	0.618697	0.467229	0.145*	0.643 (16)
O1D	0.874 (3)	0.6780 (17)	0.3906 (16)	0.093 (6)	0.357 (16)
H1OD	0.897893	0.717883	0.356287	0.139*	0.357 (16)
C23D	0.974 (4)	0.674 (4)	0.437 (3)	0.100 (7)	0.357 (16)
H23J	0.951267	0.718179	0.474309	0.151*	0.357 (16)
H23K	1.070979	0.682050	0.404077	0.151*	0.357 (16)
H23L	0.967165	0.617307	0.464908	0.151*	0.357 (16)

### Chlorido(5,12-dimethyl-1,4,8,11-tetraazacyclotetradecane)(phenylethynyl)cobalt(III) chloride–acetonitrile–methanol (1/1/1) (1) Atomic displacement parameters (Å^2^)

**Table:**

	*U*^11^	*U*^22^	*U*^33^	*U*^12^	*U*^13^	*U*^23^
Co1A	0.0442 (5)	0.0450 (5)	0.0356 (5)	0.0034 (4)	−0.0075 (4)	−0.0158 (4)
N1A	0.050 (4)	0.069 (4)	0.044 (4)	0.015 (3)	−0.009 (3)	−0.023 (3)
N2A	0.051 (4)	0.049 (3)	0.044 (3)	0.000 (3)	0.000 (3)	−0.016 (3)
N3A	0.052 (4)	0.069 (4)	0.054 (4)	0.011 (3)	−0.011 (3)	−0.017 (3)
N4A	0.048 (3)	0.057 (4)	0.043 (3)	0.000 (3)	−0.003 (3)	−0.016 (3)
Cl1A	0.0613 (11)	0.0484 (9)	0.0394 (9)	0.0005 (8)	−0.0021 (8)	−0.0189 (7)
Cl2A	0.0762 (15)	0.0857 (16)	0.0679 (14)	−0.0093 (12)	−0.0285 (11)	−0.0280 (12)
C1A	0.038 (3)	0.049 (4)	0.036 (3)	−0.005 (3)	−0.014 (3)	−0.006 (3)
C2A	0.052 (4)	0.043 (4)	0.051 (4)	0.002 (3)	−0.013 (3)	−0.012 (3)
C3A	0.055 (4)	0.043 (4)	0.041 (4)	−0.002 (3)	−0.009 (3)	−0.015 (3)
C4A	0.044 (4)	0.062 (5)	0.046 (4)	0.001 (3)	−0.011 (3)	−0.017 (4)
C5A	0.064 (5)	0.070 (5)	0.046 (4)	0.006 (4)	−0.003 (4)	−0.024 (4)
C6A	0.070 (5)	0.072 (6)	0.047 (5)	−0.003 (4)	−0.011 (4)	−0.029 (4)
C7A	0.065 (5)	0.095 (7)	0.053 (5)	0.005 (5)	−0.024 (4)	−0.032 (5)
C8A	0.056 (5)	0.080 (6)	0.058 (5)	0.020 (4)	−0.021 (4)	−0.034 (4)
C9A	0.073 (6)	0.078 (6)	0.039 (4)	0.030 (5)	−0.015 (4)	−0.015 (4)
C10A	0.089 (6)	0.043 (4)	0.041 (4)	0.008 (4)	−0.004 (4)	−0.010 (3)
C11A	0.057 (5)	0.079 (6)	0.064 (5)	−0.010 (4)	−0.001 (4)	−0.031 (5)
C12A	0.045 (5)	0.116 (9)	0.093 (8)	−0.006 (5)	−0.015 (5)	−0.043 (7)
C13A	0.039 (4)	0.109 (8)	0.078 (6)	0.006 (4)	−0.022 (4)	−0.029 (6)
C14A	0.067 (5)	0.072 (6)	0.059 (5)	0.017 (4)	−0.023 (4)	−0.003 (4)
C15A	0.087 (6)	0.037 (4)	0.060 (5)	−0.002 (4)	−0.012 (4)	−0.002 (3)
C16A	0.061 (5)	0.085 (6)	0.049 (5)	−0.018 (4)	0.000 (4)	−0.024 (4)
C17A	0.039 (4)	0.111 (8)	0.084 (7)	−0.003 (5)	−0.010 (4)	−0.042 (6)
C18A	0.044 (4)	0.109 (8)	0.067 (6)	0.007 (5)	−0.022 (4)	−0.035 (5)
C19A	0.092 (8)	0.106 (8)	0.071 (7)	−0.036 (7)	0.007 (6)	−0.030 (6)
C20A	0.088 (7)	0.112 (8)	0.069 (6)	−0.041 (6)	0.019 (5)	−0.044 (6)
N5A	0.095 (6)	0.085 (6)	0.057 (5)	−0.016 (5)	0.009 (4)	−0.019 (4)
C21A	0.070 (6)	0.067 (5)	0.050 (5)	−0.004 (4)	0.000 (4)	−0.007 (4)
C22A	0.107 (8)	0.090 (7)	0.051 (5)	0.007 (6)	−0.021 (5)	−0.017 (5)
O1A	0.084 (8)	0.110 (9)	0.103 (11)	0.016 (7)	−0.010 (8)	−0.020 (9)
C23A	0.100 (13)	0.119 (12)	0.109 (13)	0.001 (10)	−0.012 (11)	−0.009 (11)
O1C	0.090 (12)	0.105 (11)	0.087 (15)	0.011 (10)	−0.006 (11)	−0.045 (11)
C23C	0.102 (14)	0.122 (13)	0.112 (14)	0.011 (12)	−0.017 (13)	−0.029 (13)
Co2B	0.0503 (5)	0.0451 (5)	0.0300 (5)	−0.0036 (4)	−0.0080 (4)	−0.0149 (4)
N1B	0.044 (3)	0.064 (4)	0.033 (3)	−0.007 (3)	−0.004 (2)	−0.024 (3)
N2B	0.057 (4)	0.052 (3)	0.036 (3)	−0.009 (3)	−0.014 (3)	−0.014 (3)
N3B	0.057 (4)	0.061 (4)	0.047 (4)	−0.013 (3)	−0.009 (3)	−0.009 (3)
N4B	0.055 (4)	0.057 (4)	0.033 (3)	−0.004 (3)	−0.011 (3)	−0.017 (3)
Cl1B	0.0808 (13)	0.0478 (10)	0.0325 (8)	−0.0022 (9)	−0.0137 (8)	−0.0183 (7)
Cl2B	0.0600 (12)	0.0761 (14)	0.0628 (13)	0.0108 (10)	−0.0052 (10)	−0.0210 (11)
C1B	0.031 (3)	0.046 (4)	0.035 (3)	−0.001 (3)	−0.004 (3)	−0.002 (3)
C2B	0.048 (4)	0.052 (4)	0.034 (4)	−0.003 (3)	−0.008 (3)	−0.010 (3)
C3B	0.052 (4)	0.043 (4)	0.039 (4)	0.006 (3)	−0.006 (3)	−0.016 (3)
C4B	0.052 (4)	0.070 (5)	0.043 (4)	0.006 (4)	−0.014 (3)	−0.019 (4)
C5B	0.085 (6)	0.071 (6)	0.050 (5)	0.006 (5)	−0.029 (4)	−0.026 (4)
C6B	0.080 (6)	0.074 (6)	0.038 (4)	0.019 (5)	−0.013 (4)	−0.025 (4)
C7B	0.061 (5)	0.096 (7)	0.040 (4)	0.012 (5)	0.001 (4)	−0.022 (4)
C8B	0.055 (5)	0.073 (5)	0.042 (4)	0.000 (4)	−0.008 (3)	−0.015 (4)
C9B	0.060 (5)	0.067 (5)	0.033 (4)	−0.010 (4)	−0.004 (3)	−0.020 (4)
C10B	0.082 (6)	0.040 (4)	0.035 (4)	−0.017 (4)	−0.009 (4)	−0.009 (3)
C11B	0.069 (5)	0.065 (5)	0.047 (5)	0.006 (4)	−0.020 (4)	−0.012 (4)
C12B	0.057 (5)	0.080 (6)	0.079 (6)	−0.002 (4)	−0.020 (4)	−0.018 (5)
C13B	0.038 (4)	0.084 (6)	0.086 (7)	−0.006 (4)	−0.013 (4)	−0.012 (5)
C14B	0.060 (5)	0.065 (5)	0.056 (5)	−0.019 (4)	−0.012 (4)	−0.009 (4)
C15B	0.081 (6)	0.044 (4)	0.048 (4)	−0.019 (4)	−0.015 (4)	−0.001 (3)
C16B	0.062 (5)	0.065 (5)	0.050 (5)	0.005 (4)	−0.015 (4)	−0.019 (4)
C17B	0.055 (5)	0.080 (6)	0.071 (6)	0.012 (4)	−0.025 (4)	−0.021 (5)
C18B	0.037 (4)	0.081 (6)	0.073 (6)	−0.003 (4)	−0.009 (4)	−0.027 (5)
C19B	0.088 (7)	0.069 (6)	0.065 (6)	0.001 (5)	−0.032 (5)	−0.017 (5)
C20B	0.099 (8)	0.075 (6)	0.058 (6)	0.013 (5)	−0.030 (5)	−0.019 (5)
N5B	0.161 (10)	0.074 (5)	0.052 (5)	0.019 (6)	−0.035 (5)	−0.018 (4)
C21B	0.093 (7)	0.060 (5)	0.053 (5)	0.008 (5)	−0.025 (5)	−0.013 (4)
C22B	0.120 (9)	0.101 (8)	0.050 (6)	0.032 (7)	−0.017 (6)	−0.030 (6)
O1B	0.075 (8)	0.102 (8)	0.066 (7)	−0.025 (6)	−0.020 (6)	−0.011 (6)
C23B	0.086 (12)	0.112 (10)	0.088 (9)	−0.036 (11)	−0.013 (9)	−0.012 (9)
O1D	0.096 (13)	0.111 (11)	0.075 (11)	−0.025 (11)	−0.025 (9)	−0.018 (10)
C23D	0.094 (15)	0.115 (12)	0.082 (11)	−0.022 (14)	−0.005 (11)	−0.006 (11)

### Chlorido(5,12-dimethyl-1,4,8,11-tetraazacyclotetradecane)(phenylethynyl)cobalt(III) chloride–acetonitrile–methanol (1/1/1) (1) Geometric parameters (Å, º)

**Table:**

Co1A—C1A	1.893 (7)	Co2B—C1B	1.905 (7)
Co1A—N3A	1.968 (7)	Co2B—N1B	1.960 (6)
Co1A—N1A	1.973 (7)	Co2B—N3B	1.960 (7)
Co1A—N2A	1.979 (6)	Co2B—N2B	1.996 (7)
Co1A—N4A	1.982 (7)	Co2B—N4B	1.999 (7)
Co1A—Cl1A	2.3270 (18)	Co2B—Cl1B	2.3233 (18)
N1A—C18A	1.482 (11)	N1B—C9B	1.475 (10)
N1A—C9A	1.484 (11)	N1B—C18B	1.484 (11)
N1A—H1NA	1.0000	N1B—H1BN	1.0000
N2A—C11A	1.502 (11)	N2B—C10B	1.485 (9)
N2A—C10A	1.506 (11)	N2B—C11B	1.517 (11)
N2A—H2NA	1.0000	N2B—H2BN	1.0000
N3A—C13A	1.485 (11)	N3B—C14B	1.469 (11)
N3A—C14A	1.489 (12)	N3B—C13B	1.493 (11)
N3A—H3N	1.0000	N3B—H3BN	1.0000
N4A—C15A	1.487 (11)	N4B—C15B	1.487 (10)
N4A—C16A	1.512 (11)	N4B—C16B	1.514 (10)
N4A—H4N	1.0000	N4B—H4BN	1.0000
C1A—C2A	1.189 (10)	C1B—C2B	1.168 (9)
C2A—C3A	1.444 (10)	C2B—C3B	1.437 (9)
C3A—C8A	1.392 (11)	C3B—C4B	1.390 (11)
C3A—C4A	1.395 (10)	C3B—C8B	1.390 (11)
C4A—C5A	1.374 (11)	C4B—C5B	1.392 (11)
C4A—H4A	0.9500	C4B—H4B	0.9500
C5A—C6A	1.394 (13)	C5B—C6B	1.363 (13)
C5A—H5A	0.9500	C5B—H5B	0.9500
C6A—C7A	1.368 (12)	C6B—C7B	1.387 (13)
C6A—H6A	0.9500	C6B—H6B	0.9500
C7A—C8A	1.371 (11)	C7B—C8B	1.393 (11)
C7A—H7A	0.9500	C7B—H7B	0.9500
C8A—H8A	0.9500	C8B—H8B	0.9500
C9A—C10A	1.486 (13)	C9B—C10B	1.510 (12)
C9A—H9A	0.9900	C9B—H9C	0.9900
C9A—H9B	0.9900	C9B—H9D	0.9900
C10A—H10A	0.9900	C10B—H10C	0.9900
C10A—H10B	0.9900	C10B—H10D	0.9900
C11A—C19A	1.509 (14)	C11B—C12B	1.509 (13)
C11A—C12A	1.539 (15)	C11B—C19B	1.521 (12)
C11A—H11A	1.0000	C11B—H11B	1.0000
C12A—C13A	1.499 (15)	C12B—C13B	1.477 (14)
C12A—H12A	0.9900	C12B—H12C	0.9900
C12A—H12B	0.9900	C12B—H12D	0.9900
C13A—H13A	0.9900	C13B—H13C	0.9900
C13A—H13B	0.9900	C13B—H13D	0.9900
C14A—C15A	1.489 (13)	C14B—C15B	1.495 (13)
C14A—H14A	0.9900	C14B—H14C	0.9900
C14A—H14B	0.9900	C14B—H14D	0.9900
C15A—H15A	0.9900	C15B—H15C	0.9900
C15A—H15B	0.9900	C15B—H15D	0.9900
C16A—C20A	1.517 (13)	C16B—C17B	1.497 (12)
C16A—C17A	1.526 (15)	C16B—C20B	1.528 (13)
C16A—H16A	1.0000	C16B—H16B	1.0000
C17A—C18A	1.456 (14)	C17B—C18B	1.488 (13)
C17A—H17A	0.9900	C17B—H17C	0.9900
C17A—H17B	0.9900	C17B—H17D	0.9900
C18A—H18A	0.9900	C18B—H18C	0.9900
C18A—H18B	0.9900	C18B—H18D	0.9900
C19A—H19A	0.9800	C19B—H19D	0.9800
C19A—H19B	0.9800	C19B—H19E	0.9800
C19A—H19C	0.9800	C19B—H19F	0.9800
C20A—H20A	0.9800	C20B—H20D	0.9800
C20A—H20B	0.9800	C20B—H20E	0.9800
C20A—H20C	0.9800	C20B—H20F	0.9800
N5A—C21A	1.128 (12)	N5B—C21B	1.118 (11)
C21A—C22A	1.454 (12)	C21B—C22B	1.444 (12)
C22A—H22A	0.9800	C22B—H22D	0.9800
C22A—H22B	0.9800	C22B—H22E	0.9800
C22A—H22C	0.9800	C22B—H22F	0.9800
O1A—C23A	1.378 (17)	O1B—C23B	1.416 (17)
O1A—H1OA	0.8400	O1B—H1OB	0.8400
C23A—H23A	0.9800	C23B—H23D	0.9800
C23A—H23B	0.9800	C23B—H23E	0.9800
C23A—H23C	0.9800	C23B—H23F	0.9800
O1C—C23C	1.421 (19)	O1D—C23D	1.430 (19)
O1C—H1OC	0.8400	O1D—H1OD	0.8400
C23C—H23G	0.9800	C23D—H23J	0.9800
C23C—H23H	0.9800	C23D—H23K	0.9800
C23C—H23I	0.9800	C23D—H23L	0.9800
			
C1A—Co1A—N3A	89.8 (3)	C1B—Co2B—N1B	89.9 (2)
C1A—Co1A—N1A	89.6 (3)	C1B—Co2B—N3B	89.3 (3)
N3A—Co1A—N1A	179.1 (3)	N1B—Co2B—N3B	179.3 (3)
C1A—Co1A—N2A	91.7 (3)	C1B—Co2B—N2B	92.2 (3)
N3A—Co1A—N2A	94.4 (3)	N1B—Co2B—N2B	86.7 (3)
N1A—Co1A—N2A	86.2 (3)	N3B—Co2B—N2B	93.4 (3)
C1A—Co1A—N4A	88.2 (3)	C1B—Co2B—N4B	88.0 (3)
N3A—Co1A—N4A	85.6 (3)	N1B—Co2B—N4B	92.9 (3)
N1A—Co1A—N4A	93.9 (3)	N3B—Co2B—N4B	87.1 (3)
N2A—Co1A—N4A	179.8 (3)	N2B—Co2B—N4B	179.5 (3)
C1A—Co1A—Cl1A	177.7 (2)	C1B—Co2B—Cl1B	178.0 (2)
N3A—Co1A—Cl1A	88.0 (2)	N1B—Co2B—Cl1B	92.08 (17)
N1A—Co1A—Cl1A	92.66 (18)	N3B—Co2B—Cl1B	88.6 (2)
N2A—Co1A—Cl1A	88.08 (18)	N2B—Co2B—Cl1B	87.81 (18)
N4A—Co1A—Cl1A	92.09 (18)	N4B—Co2B—Cl1B	92.00 (17)
C18A—N1A—C9A	110.3 (7)	C9B—N1B—C18B	112.2 (6)
C18A—N1A—Co1A	118.2 (6)	C9B—N1B—Co2B	107.6 (5)
C9A—N1A—Co1A	108.0 (5)	C18B—N1B—Co2B	118.8 (5)
C18A—N1A—H1NA	106.5	C9B—N1B—H1BN	105.8
C9A—N1A—H1NA	106.5	C18B—N1B—H1BN	105.8
Co1A—N1A—H1NA	106.5	Co2B—N1B—H1BN	105.8
C11A—N2A—C10A	111.0 (7)	C10B—N2B—C11B	110.8 (6)
C11A—N2A—Co1A	119.2 (6)	C10B—N2B—Co2B	107.1 (5)
C10A—N2A—Co1A	107.4 (5)	C11B—N2B—Co2B	120.2 (5)
C11A—N2A—H2NA	106.1	C10B—N2B—H2BN	105.9
C10A—N2A—H2NA	106.1	C11B—N2B—H2BN	105.9
Co1A—N2A—H2NA	106.1	Co2B—N2B—H2BN	105.9
C13A—N3A—C14A	109.1 (7)	C14B—N3B—C13B	112.0 (7)
C13A—N3A—Co1A	118.1 (6)	C14B—N3B—Co2B	107.7 (5)
C14A—N3A—Co1A	108.5 (5)	C13B—N3B—Co2B	118.4 (5)
C13A—N3A—H3N	106.9	C14B—N3B—H3BN	106.0
C14A—N3A—H3N	106.9	C13B—N3B—H3BN	106.0
Co1A—N3A—H3N	106.9	Co2B—N3B—H3BN	106.0
C15A—N4A—C16A	111.1 (7)	C15B—N4B—C16B	111.9 (6)
C15A—N4A—Co1A	108.0 (5)	C15B—N4B—Co2B	106.4 (5)
C16A—N4A—Co1A	118.6 (5)	C16B—N4B—Co2B	120.5 (5)
C15A—N4A—H4N	106.1	C15B—N4B—H4BN	105.7
C16A—N4A—H4N	106.1	C16B—N4B—H4BN	105.7
Co1A—N4A—H4N	106.1	Co2B—N4B—H4BN	105.7
C2A—C1A—Co1A	171.3 (7)	C2B—C1B—Co2B	171.8 (6)
C1A—C2A—C3A	175.3 (8)	C1B—C2B—C3B	176.3 (8)
C8A—C3A—C4A	118.1 (7)	C4B—C3B—C8B	118.1 (7)
C8A—C3A—C2A	119.8 (7)	C4B—C3B—C2B	121.9 (7)
C4A—C3A—C2A	122.0 (7)	C8B—C3B—C2B	120.0 (7)
C5A—C4A—C3A	120.1 (8)	C3B—C4B—C5B	120.5 (8)
C5A—C4A—H4A	119.9	C3B—C4B—H4B	119.7
C3A—C4A—H4A	119.9	C5B—C4B—H4B	119.7
C4A—C5A—C6A	120.9 (8)	C6B—C5B—C4B	120.9 (8)
C4A—C5A—H5A	119.5	C6B—C5B—H5B	119.5
C6A—C5A—H5A	119.5	C4B—C5B—H5B	119.5
C7A—C6A—C5A	118.9 (7)	C5B—C6B—C7B	119.5 (7)
C7A—C6A—H6A	120.5	C5B—C6B—H6B	120.2
C5A—C6A—H6A	120.5	C7B—C6B—H6B	120.2
C6A—C7A—C8A	120.6 (8)	C6B—C7B—C8B	119.8 (8)
C6A—C7A—H7A	119.7	C6B—C7B—H7B	120.1
C8A—C7A—H7A	119.7	C8B—C7B—H7B	120.1
C7A—C8A—C3A	121.3 (8)	C3B—C8B—C7B	121.1 (8)
C7A—C8A—H8A	119.4	C3B—C8B—H8B	119.5
C3A—C8A—H8A	119.4	C7B—C8B—H8B	119.5
N1A—C9A—C10A	107.7 (7)	N1B—C9B—C10B	108.2 (6)
N1A—C9A—H9A	110.2	N1B—C9B—H9C	110.0
C10A—C9A—H9A	110.2	C10B—C9B—H9C	110.0
N1A—C9A—H9B	110.2	N1B—C9B—H9D	110.0
C10A—C9A—H9B	110.2	C10B—C9B—H9D	110.0
H9A—C9A—H9B	108.5	H9C—C9B—H9D	108.4
C9A—C10A—N2A	107.1 (6)	N2B—C10B—C9B	106.6 (6)
C9A—C10A—H10A	110.3	N2B—C10B—H10C	110.4
N2A—C10A—H10A	110.3	C9B—C10B—H10C	110.4
C9A—C10A—H10B	110.3	N2B—C10B—H10D	110.4
N2A—C10A—H10B	110.3	C9B—C10B—H10D	110.4
H10A—C10A—H10B	108.5	H10C—C10B—H10D	108.6
N2A—C11A—C19A	111.7 (8)	C12B—C11B—N2B	109.8 (7)
N2A—C11A—C12A	110.4 (7)	C12B—C11B—C19B	109.4 (8)
C19A—C11A—C12A	110.4 (9)	N2B—C11B—C19B	112.7 (7)
N2A—C11A—H11A	108.1	C12B—C11B—H11B	108.3
C19A—C11A—H11A	108.1	N2B—C11B—H11B	108.3
C12A—C11A—H11A	108.1	C19B—C11B—H11B	108.3
C13A—C12A—C11A	115.4 (8)	C13B—C12B—C11B	115.2 (8)
C13A—C12A—H12A	108.4	C13B—C12B—H12C	108.5
C11A—C12A—H12A	108.4	C11B—C12B—H12C	108.5
C13A—C12A—H12B	108.4	C13B—C12B—H12D	108.5
C11A—C12A—H12B	108.4	C11B—C12B—H12D	108.5
H12A—C12A—H12B	107.5	H12C—C12B—H12D	107.5
N3A—C13A—C12A	109.9 (8)	C12B—C13B—N3B	112.7 (8)
N3A—C13A—H13A	109.7	C12B—C13B—H13C	109.1
C12A—C13A—H13A	109.7	N3B—C13B—H13C	109.1
N3A—C13A—H13B	109.7	C12B—C13B—H13D	109.1
C12A—C13A—H13B	109.7	N3B—C13B—H13D	109.1
H13A—C13A—H13B	108.2	H13C—C13B—H13D	107.8
C15A—C14A—N3A	106.9 (7)	N3B—C14B—C15B	108.8 (7)
C15A—C14A—H14A	110.3	N3B—C14B—H14C	109.9
N3A—C14A—H14A	110.3	C15B—C14B—H14C	109.9
C15A—C14A—H14B	110.3	N3B—C14B—H14D	109.9
N3A—C14A—H14B	110.3	C15B—C14B—H14D	109.9
H14A—C14A—H14B	108.6	H14C—C14B—H14D	108.3
N4A—C15A—C14A	106.9 (6)	N4B—C15B—C14B	108.2 (6)
N4A—C15A—H15A	110.3	N4B—C15B—H15C	110.1
C14A—C15A—H15A	110.3	C14B—C15B—H15C	110.1
N4A—C15A—H15B	110.3	N4B—C15B—H15D	110.1
C14A—C15A—H15B	110.3	C14B—C15B—H15D	110.1
H15A—C15A—H15B	108.6	H15C—C15B—H15D	108.4
N4A—C16A—C20A	111.4 (8)	C17B—C16B—N4B	108.5 (7)
N4A—C16A—C17A	110.4 (7)	C17B—C16B—C20B	111.4 (8)
C20A—C16A—C17A	111.6 (9)	N4B—C16B—C20B	112.2 (7)
N4A—C16A—H16A	107.7	C17B—C16B—H16B	108.2
C20A—C16A—H16A	107.7	N4B—C16B—H16B	108.2
C17A—C16A—H16A	107.7	C20B—C16B—H16B	108.2
C18A—C17A—C16A	116.3 (8)	C18B—C17B—C16B	115.3 (7)
C18A—C17A—H17A	108.2	C18B—C17B—H17C	108.5
C16A—C17A—H17A	108.2	C16B—C17B—H17C	108.5
C18A—C17A—H17B	108.2	C18B—C17B—H17D	108.5
C16A—C17A—H17B	108.2	C16B—C17B—H17D	108.5
H17A—C17A—H17B	107.4	H17C—C17B—H17D	107.5
C17A—C18A—N1A	111.4 (7)	N1B—C18B—C17B	113.5 (7)
C17A—C18A—H18A	109.4	N1B—C18B—H18C	108.9
N1A—C18A—H18A	109.4	C17B—C18B—H18C	108.9
C17A—C18A—H18B	109.4	N1B—C18B—H18D	108.9
N1A—C18A—H18B	109.4	C17B—C18B—H18D	108.9
H18A—C18A—H18B	108.0	H18C—C18B—H18D	107.7
C11A—C19A—H19A	109.5	C11B—C19B—H19D	109.5
C11A—C19A—H19B	109.5	C11B—C19B—H19E	109.5
H19A—C19A—H19B	109.5	H19D—C19B—H19E	109.5
C11A—C19A—H19C	109.5	C11B—C19B—H19F	109.5
H19A—C19A—H19C	109.5	H19D—C19B—H19F	109.5
H19B—C19A—H19C	109.5	H19E—C19B—H19F	109.5
C16A—C20A—H20A	109.5	C16B—C20B—H20D	109.5
C16A—C20A—H20B	109.5	C16B—C20B—H20E	109.5
H20A—C20A—H20B	109.5	H20D—C20B—H20E	109.5
C16A—C20A—H20C	109.5	C16B—C20B—H20F	109.5
H20A—C20A—H20C	109.5	H20D—C20B—H20F	109.5
H20B—C20A—H20C	109.5	H20E—C20B—H20F	109.5
N5A—C21A—C22A	179.0 (13)	N5B—C21B—C22B	179.7 (14)
C21A—C22A—H22A	109.5	C21B—C22B—H22D	109.5
C21A—C22A—H22B	109.5	C21B—C22B—H22E	109.5
H22A—C22A—H22B	109.5	H22D—C22B—H22E	109.5
C21A—C22A—H22C	109.5	C21B—C22B—H22F	109.5
H22A—C22A—H22C	109.5	H22D—C22B—H22F	109.5
H22B—C22A—H22C	109.5	H22E—C22B—H22F	109.5
C23A—O1A—H1OA	109.5	C23B—O1B—H1OB	109.5
O1A—C23A—H23A	109.5	O1B—C23B—H23D	109.5
O1A—C23A—H23B	109.5	O1B—C23B—H23E	109.5
H23A—C23A—H23B	109.5	H23D—C23B—H23E	109.5
O1A—C23A—H23C	109.5	O1B—C23B—H23F	109.5
H23A—C23A—H23C	109.5	H23D—C23B—H23F	109.5
H23B—C23A—H23C	109.5	H23E—C23B—H23F	109.5
C23C—O1C—H1OC	109.5	C23D—O1D—H1OD	109.5
O1C—C23C—H23G	109.5	O1D—C23D—H23J	109.5
O1C—C23C—H23H	109.5	O1D—C23D—H23K	109.5
H23G—C23C—H23H	109.5	H23J—C23D—H23K	109.5
O1C—C23C—H23I	109.5	O1D—C23D—H23L	109.5
H23G—C23C—H23I	109.5	H23J—C23D—H23L	109.5
H23H—C23C—H23I	109.5	H23K—C23D—H23L	109.5
			
C8A—C3A—C4A—C5A	0.8 (12)	C8B—C3B—C4B—C5B	−0.3 (13)
C2A—C3A—C4A—C5A	−179.7 (8)	C2B—C3B—C4B—C5B	−179.4 (8)
C3A—C4A—C5A—C6A	−0.8 (13)	C3B—C4B—C5B—C6B	−2.0 (14)
C4A—C5A—C6A—C7A	0.9 (14)	C4B—C5B—C6B—C7B	3.2 (15)
C5A—C6A—C7A—C8A	−1.0 (15)	C5B—C6B—C7B—C8B	−2.1 (15)
C6A—C7A—C8A—C3A	0.9 (16)	C4B—C3B—C8B—C7B	1.4 (13)
C4A—C3A—C8A—C7A	−0.8 (14)	C2B—C3B—C8B—C7B	−179.5 (8)
C2A—C3A—C8A—C7A	179.7 (9)	C6B—C7B—C8B—C3B	−0.2 (15)
C18A—N1A—C9A—C10A	171.1 (7)	C18B—N1B—C9B—C10B	−173.1 (6)
Co1A—N1A—C9A—C10A	40.5 (7)	Co2B—N1B—C9B—C10B	−40.7 (6)
N1A—C9A—C10A—N2A	−53.2 (8)	C11B—N2B—C10B—C9B	−172.6 (6)
C11A—N2A—C10A—C9A	172.0 (6)	Co2B—N2B—C10B—C9B	−39.7 (6)
Co1A—N2A—C10A—C9A	40.1 (7)	N1B—C9B—C10B—N2B	53.7 (7)
C10A—N2A—C11A—C19A	59.0 (9)	C10B—N2B—C11B—C12B	178.7 (7)
Co1A—N2A—C11A—C19A	−175.4 (7)	Co2B—N2B—C11B—C12B	52.9 (8)
C10A—N2A—C11A—C12A	−177.7 (7)	C10B—N2B—C11B—C19B	−59.0 (8)
Co1A—N2A—C11A—C12A	−52.2 (8)	Co2B—N2B—C11B—C19B	175.2 (5)
N2A—C11A—C12A—C13A	66.8 (10)	N2B—C11B—C12B—C13B	−66.2 (10)
C19A—C11A—C12A—C13A	−169.2 (8)	C19B—C11B—C12B—C13B	169.6 (8)
C14A—N3A—C13A—C12A	−176.3 (8)	C11B—C12B—C13B—N3B	69.7 (11)
Co1A—N3A—C13A—C12A	59.3 (9)	C14B—N3B—C13B—C12B	176.9 (7)
C11A—C12A—C13A—N3A	−70.5 (10)	Co2B—N3B—C13B—C12B	−56.9 (9)
C13A—N3A—C14A—C15A	−170.5 (7)	C13B—N3B—C14B—C15B	171.3 (7)
Co1A—N3A—C14A—C15A	−40.6 (8)	Co2B—N3B—C14B—C15B	39.4 (7)
C16A—N4A—C15A—C14A	−172.8 (7)	C16B—N4B—C15B—C14B	171.6 (6)
Co1A—N4A—C15A—C14A	−41.1 (8)	Co2B—N4B—C15B—C14B	38.1 (7)
N3A—C14A—C15A—N4A	53.6 (9)	N3B—C14B—C15B—N4B	−52.2 (8)
C15A—N4A—C16A—C20A	−56.7 (9)	C15B—N4B—C16B—C17B	178.5 (7)
Co1A—N4A—C16A—C20A	177.4 (6)	Co2B—N4B—C16B—C17B	−55.4 (8)
C15A—N4A—C16A—C17A	178.7 (7)	C15B—N4B—C16B—C20B	55.0 (9)
Co1A—N4A—C16A—C17A	52.7 (8)	Co2B—N4B—C16B—C20B	−178.9 (5)
N4A—C16A—C17A—C18A	−66.7 (10)	N4B—C16B—C17B—C18B	67.2 (9)
C20A—C16A—C17A—C18A	168.8 (8)	C20B—C16B—C17B—C18B	−168.8 (7)
C16A—C17A—C18A—N1A	69.5 (10)	C9B—N1B—C18B—C17B	−178.8 (7)
C9A—N1A—C18A—C17A	177.3 (7)	Co2B—N1B—C18B—C17B	54.7 (8)
Co1A—N1A—C18A—C17A	−57.7 (9)	C16B—C17B—C18B—N1B	−69.0 (10)

### Chlorido(5,12-dimethyl-1,4,8,11-tetraazacyclotetradecane)(phenylethynyl)cobalt(III) chloride–acetonitrile–methanol (1/1/1) (1) Hydrogen-bond geometry (Å, º)

**Table:**

*D*—H···*A*	*D*—H	H···*A*	*D*···*A*	*D*—H···*A*
N1*A*—H1*NA*···Cl2*B*^i^	1.00	2.40	3.265 (6)	144
N2*A*—H2*NA*···Cl1*A*^ii^	1.00	2.91	3.684 (7)	134
N3*A*—H3*N*···O1*A*	1.00	2.02	2.844 (16)	138
N3*A*—H3*N*···O1*C*	1.00	2.14	3.10 (3)	160
N4*A*—H4*N*···N5*A*	1.00	2.30	3.185 (10)	147
N1*B*—H1*BN*···Cl2*A*	1.00	2.40	3.251 (6)	143
N2*B*—H2*BN*···Cl1*B*^iii^	1.00	2.83	3.607 (6)	135
N3*B*—H3*BN*···O1*B*	1.00	2.06	2.863 (13)	136
N3*B*—H3*BN*···O1*D*	1.00	2.08	3.03 (3)	158
N4*B*—H4*BN*···N5*B*	1.00	2.26	3.148 (10)	147
O1*A*—H1*OA*···Cl2*A*	0.84	2.09	2.899 (18)	163
O1*B*—H1*OB*···Cl2*B*	0.84	2.19	2.979 (13)	157
O1*C*—H1*OC*···Cl2*A*	0.84	2.40	3.16 (4)	152
O1*D*—H1*OD*···Cl2*B*	0.84	2.30	3.13 (3)	168
C9*A*—H9*A*···Cl1*A*^ii^	0.99	2.92	3.631 (9)	130
C10*A*—H10*A*···Cl1*A*^ii^	0.99	2.96	3.531 (8)	118
C14*A*—H14*B*···Cl2*A*	0.99	2.98	3.884 (10)	152
C16*A*—H16*A*···Cl1*A*	1.00	2.81	3.373 (9)	116
C17*A*—H17*B*···O1*A*^iv^	0.99	2.46	3.44 (2)	169
C18*A*—H18*A*···Cl1*A*	0.99	2.78	3.339 (9)	116
C20*A*—H20*B*···Cl2*A*^iv^	0.98	2.88	3.834 (11)	164
C22*A*—H22*A*···Cl2*B*^v^	0.98	2.82	3.669 (11)	146
C9*B*—H9*C*···Cl2*A*	0.99	2.97	3.498 (8)	114
C10*B*—H10*C*···Cl1*B*^iii^	0.99	2.89	3.485 (7)	120
C16*B*—H16*B*···Cl1*B*	1.00	2.85	3.407 (9)	116
C17*B*—H17*C*···O1*B*^vi^	0.99	2.63	3.479 (18)	144
C18*B*—H18*C*···Cl1*B*	0.99	2.83	3.358 (9)	114
C22*B*—H22*E*···Cl2*A*^vii^	0.98	2.81	3.583 (12)	137

Symmetry codes: (i) *x*, *y*−1, *z*; (ii) −*x*+1, −*y*, −*z*+1; (iii) −*x*+1, −*y*+1, −*z*+1; (iv) *x*+1, *y*, *z*; (v) −*x*+2, −*y*+1, −*z*; (vi) *x*−1, *y*, *z*; (vii) −*x*+1, −*y*+1, −*z*.

### (5,12-Dimethyl-1,4,8,11-tetraazacyclotetradecane)bis(phenylethynyl)cobalt(III) trifluoromethanesulfonate–dichloromethane (2/1) (2) Crystal data

**Table:**

2[Co(C_8_H_5_)_2_(C_12_H_28_N_4_)](CF_3_SO_3_)_2_·CH_2_Cl_2_	*F*(000) = 1420
*M**_r_* = 1362.17	*D*_x_ = 1.435 Mg m^−^^3^
Monoclinic, *P*2_1_	Mo *K*α radiation, λ = 0.71073 Å
*a* = 12.0263 (7) Å	Cell parameters from 9895 reflections
*b* = 12.3999 (5) Å	θ = 3.3–32.9°
*c* = 21.9164 (14) Å	µ = 0.75 mm^−^^1^
β = 105.3260 (14)°	*T* = 150 K
*V* = 3152.1 (3) Å^3^	Plate, yellow
*Z* = 2	0.40 × 0.30 × 0.10 mm

### (5,12-Dimethyl-1,4,8,11-tetraazacyclotetradecane)bis(phenylethynyl)cobalt(III) trifluoromethanesulfonate–dichloromethane (2/1) (2) Data collection

**Table:**

Bruker AXS D8 Quest CMOS diffractometer	22462 independent reflections
Radiation source: sealed tube X-ray source	18066 reflections with *I* > 2σ(*I*)
Triumph curved graphite crystal monochromator	*R*_int_ = 0.026
ω and phi scans	θ_max_ = 33.2°, θ_min_ = 2.9°
Absorption correction: multi-scan (SADABS; Krause et al., 2015)	*h* = −18→18
*T*_min_ = 0.660, *T*_max_ = 0.747	*k* = −19→17
54456 measured reflections	*l* = −30→33

### (5,12-Dimethyl-1,4,8,11-tetraazacyclotetradecane)bis(phenylethynyl)cobalt(III) trifluoromethanesulfonate–dichloromethane (2/1) (2) Refinement

**Table:**

Refinement on *F*^2^	Hydrogen site location: inferred from neighbouring sites
Least-squares matrix: full	H-atom parameters constrained
*R*[*F*^2^ > 2σ(*F*^2^)] = 0.034	*w* = 1/[σ^2^(*F*_o_^2^) + (0.0368*P*)^2^] where *P* = (*F*_o_^2^ + 2*F*_c_^2^)/3
*wR*(*F*^2^) = 0.078	(Δ/σ)_max_ = 0.001
*S* = 1.01	Δρ_max_ = 0.44 e Å^−^^3^
22462 reflections	Δρ_min_ = −0.56 e Å^−^^3^
872 parameters	Extinction correction: SHELXL2018 (Sheldrick, 2015), Fc^*^=kFc[1+0.001xFc^2^λ^3^/sin(2θ)]^-1/4^
349 restraints	Extinction coefficient: 0.0043 (5)
Primary atom site location: structure-invariant direct methods	Absolute structure: Flack *x* determined using 6987 quotients [(*I*^+^)-(*I*^-^)]/[(*I*^+^)+(*I*^-^)] (Parsons et al., 2013)
Secondary atom site location: difference Fourier map	Absolute structure parameter: −0.003 (3)

### (5,12-Dimethyl-1,4,8,11-tetraazacyclotetradecane)bis(phenylethynyl)cobalt(III) trifluoromethanesulfonate–dichloromethane (2/1) (2) Special details

**Table:**

Geometry. All esds (except the esd in the dihedral angle between two l.s. planes) are estimated using the full covariance matrix. The cell esds are taken into account individually in the estimation of esds in distances, angles and torsion angles; correlations between esds in cell parameters are only used when they are defined by crystal symmetry. An approximate (isotropic) treatment of cell esds is used for estimating esds involving l.s. planes.
Refinement. The S1 triflate anion was refined as two-component disorder. Each moiety was restrained to have to same geometries as the S2 triflate anion. The Uij components for atoms within 2.0 Angstrom were restrained to be similar. Subject to these conditions, the occupancy factors refined to 0.503 (22) and 0.497 (22).The dichloromethane molecule was refined as two-component disorder. The minor moiety was restrained to have the same geometries as the major moiety. The Uij components for atoms within 2.0 Angstrom were restrained to be similar. Subject to these conditions, the occupancy factors refined to 0.545 (12) and 0.455 (12).

### (5,12-Dimethyl-1,4,8,11-tetraazacyclotetradecane)bis(phenylethynyl)cobalt(III) trifluoromethanesulfonate–dichloromethane (2/1) (2) Fractional atomic coordinates and isotropic or equivalent isotropic displacement parameters (Å^2^)

**Table:**

	*x*	*y*	*z*	*U*_iso_*/*U*_eq_	Occ. (<1)
Co1	0.22599 (2)	0.74080 (2)	0.48710 (2)	0.01937 (6)	
N1	0.17596 (16)	0.88594 (16)	0.50613 (9)	0.0250 (4)	
H1N	0.202133	0.936824	0.477355	0.030*	
N2	0.06447 (15)	0.72137 (15)	0.43196 (8)	0.0259 (4)	
H2N	0.022958	0.677507	0.457303	0.031*	
N3	0.27611 (17)	0.59624 (16)	0.46631 (10)	0.0293 (4)	
H3N	0.248908	0.543705	0.493847	0.035*	
N4	0.38659 (15)	0.75942 (16)	0.54171 (9)	0.0273 (4)	
H4N	0.429277	0.802430	0.516602	0.033*	
C1	0.18010 (17)	0.67531 (17)	0.55641 (10)	0.0221 (4)	
C2	0.15508 (18)	0.62530 (17)	0.59859 (10)	0.0233 (4)	
C3	0.12824 (17)	0.56768 (17)	0.64983 (9)	0.0222 (4)	
C4	0.0332 (2)	0.5971 (2)	0.67176 (11)	0.0295 (5)	
H4	−0.014296	0.655535	0.652466	0.035*	
C5	0.0077 (2)	0.5419 (3)	0.72122 (11)	0.0381 (5)	
H5	−0.057567	0.562021	0.735349	0.046*	
C6	0.0764 (2)	0.4581 (3)	0.74996 (11)	0.0428 (7)	
H6	0.058562	0.420360	0.783891	0.051*	
C7	0.1710 (2)	0.4285 (2)	0.72980 (11)	0.0396 (6)	
H7	0.218782	0.371064	0.750254	0.048*	
C8	0.19740 (19)	0.4824 (2)	0.67952 (11)	0.0302 (5)	
H8	0.262372	0.460982	0.665481	0.036*	
C9	0.27097 (17)	0.80612 (19)	0.41753 (10)	0.0245 (4)	
C10	0.29546 (19)	0.85630 (19)	0.37578 (10)	0.0269 (4)	
C11	0.32411 (19)	0.91203 (17)	0.32458 (10)	0.0249 (4)	
C12	0.2475 (2)	0.91150 (19)	0.26354 (11)	0.0295 (5)	
H12	0.175605	0.875309	0.256457	0.035*	
C13	0.2760 (2)	0.9629 (2)	0.21413 (12)	0.0367 (5)	
H13	0.223904	0.961540	0.173143	0.044*	
C14	0.3799 (3)	1.0166 (2)	0.22371 (13)	0.0398 (6)	
H14	0.398795	1.052703	0.189565	0.048*	
C15	0.4562 (3)	1.0175 (2)	0.28327 (15)	0.0434 (7)	
H15	0.527715	1.054196	0.290004	0.052*	
C16	0.4288 (2)	0.9653 (2)	0.33284 (13)	0.0353 (5)	
H16	0.482443	0.965683	0.373394	0.042*	
C17	0.04871 (19)	0.8884 (2)	0.48645 (11)	0.0298 (5)	
H17A	0.021143	0.963983	0.481517	0.036*	
H17B	0.017350	0.853221	0.518891	0.036*	
C18	0.00871 (19)	0.8294 (2)	0.42431 (11)	0.0300 (5)	
H18A	−0.076223	0.821484	0.412549	0.036*	
H18B	0.030624	0.870218	0.390447	0.036*	
C19	0.0467 (2)	0.6646 (2)	0.37000 (11)	0.0342 (5)	
H19	0.085022	0.708200	0.342979	0.041*	
C20	0.1039 (3)	0.5545 (2)	0.37961 (13)	0.0428 (6)	
H20A	0.074878	0.514498	0.411330	0.051*	
H20B	0.080063	0.514157	0.339277	0.051*	
C21	0.2337 (2)	0.5562 (2)	0.40102 (12)	0.0399 (6)	
H21A	0.263483	0.482366	0.398441	0.048*	
H21B	0.263566	0.602939	0.372306	0.048*	
C22	0.4035 (2)	0.5954 (2)	0.48753 (14)	0.0371 (6)	
H22A	0.435929	0.633284	0.456378	0.045*	
H22B	0.432306	0.520229	0.491584	0.045*	
C23	0.4389 (2)	0.6508 (2)	0.54976 (13)	0.0356 (5)	
H23A	0.411949	0.609614	0.581826	0.043*	
H23B	0.524007	0.656398	0.564058	0.043*	
C24	0.40217 (19)	0.8167 (2)	0.60386 (10)	0.0326 (5)	
H24	0.359511	0.775144	0.629613	0.039*	
C25	0.3503 (2)	0.9283 (2)	0.59300 (12)	0.0365 (5)	
H25A	0.381785	0.965599	0.561265	0.044*	
H25B	0.374679	0.969513	0.633007	0.044*	
C26	0.2197 (2)	0.9300 (2)	0.57045 (11)	0.0315 (5)	
H26A	0.187701	0.887380	0.600058	0.038*	
H26B	0.192377	1.005220	0.571076	0.038*	
C27	−0.0805 (3)	0.6548 (3)	0.33523 (13)	0.0485 (7)	
H27A	−0.088185	0.621984	0.293640	0.073*	
H27B	−0.115760	0.726632	0.329807	0.073*	
H27C	−0.119498	0.609501	0.359861	0.073*	
C28	0.5295 (2)	0.8221 (3)	0.64130 (14)	0.0497 (7)	
H28A	0.560187	0.748794	0.649729	0.075*	
H28B	0.536036	0.859537	0.681479	0.075*	
H28C	0.573378	0.861355	0.616608	0.075*	
Co2	0.23261 (2)	0.54263 (2)	0.01865 (2)	0.01757 (6)	
N5	0.29019 (15)	0.69043 (15)	0.04341 (9)	0.0244 (4)	
H5N	0.233963	0.741032	0.015845	0.029*	
N6	0.34959 (14)	0.53764 (17)	−0.03178 (8)	0.0239 (3)	
H6N	0.417814	0.498958	−0.004586	0.029*	
N7	0.17491 (15)	0.39501 (15)	−0.00536 (8)	0.0223 (3)	
H7N	0.230683	0.344403	0.022503	0.027*	
N8	0.11669 (14)	0.54874 (15)	0.06975 (7)	0.0216 (3)	
H8N	0.048813	0.589607	0.043873	0.026*	
C29	0.34839 (17)	0.48017 (17)	0.08770 (9)	0.0219 (4)	
C30	0.42740 (18)	0.43525 (18)	0.12509 (9)	0.0235 (4)	
C31	0.52463 (17)	0.38645 (18)	0.16944 (9)	0.0222 (4)	
C32	0.53286 (19)	0.27573 (19)	0.17816 (11)	0.0286 (5)	
H32	0.472746	0.230256	0.155126	0.034*	
C33	0.6291 (2)	0.2308 (2)	0.22065 (11)	0.0348 (5)	
H33	0.633967	0.154775	0.226044	0.042*	
C34	0.71686 (19)	0.2948 (2)	0.25480 (10)	0.0352 (5)	
H34	0.781918	0.263504	0.283697	0.042*	
C35	0.7094 (2)	0.4057 (2)	0.24661 (12)	0.0397 (6)	
H35	0.769803	0.450554	0.269971	0.048*	
C36	0.6140 (2)	0.4516 (2)	0.20435 (11)	0.0326 (5)	
H36	0.609529	0.527630	0.199170	0.039*	
C37	0.12161 (16)	0.60579 (17)	−0.05219 (9)	0.0208 (4)	
C38	0.05723 (17)	0.65390 (17)	−0.09550 (9)	0.0218 (4)	
C39	−0.01624 (17)	0.70998 (17)	−0.14853 (9)	0.0213 (4)	
C40	−0.13659 (19)	0.70282 (19)	−0.16176 (10)	0.0275 (4)	
H40	−0.170556	0.660721	−0.135243	0.033*	
C41	−0.2068 (2)	0.7569 (2)	−0.21348 (11)	0.0362 (5)	
H41	−0.288337	0.752055	−0.221974	0.043*	
C42	−0.1575 (2)	0.8180 (2)	−0.25253 (11)	0.0393 (6)	
H42	−0.205294	0.854604	−0.287930	0.047*	
C43	−0.0389 (2)	0.8256 (2)	−0.23998 (11)	0.0357 (5)	
H43	−0.005428	0.867340	−0.266854	0.043*	
C44	0.03152 (19)	0.77238 (18)	−0.18824 (10)	0.0274 (4)	
H44	0.112915	0.778514	−0.179796	0.033*	
C45	0.40012 (19)	0.7033 (2)	0.02585 (12)	0.0306 (5)	
H45A	0.463891	0.669858	0.058398	0.037*	
H45B	0.417709	0.780841	0.022930	0.037*	
C46	0.3882 (2)	0.6496 (2)	−0.03681 (12)	0.0302 (5)	
H46A	0.331146	0.688619	−0.070426	0.036*	
H46B	0.463140	0.649889	−0.047581	0.036*	
C47	0.32107 (19)	0.4804 (2)	−0.09431 (10)	0.0270 (4)	
H47	0.258128	0.520900	−0.124563	0.032*	
C48	0.2788 (2)	0.3669 (2)	−0.08736 (11)	0.0295 (5)	
H48A	0.337745	0.329309	−0.054003	0.035*	
H48B	0.271897	0.327716	−0.127544	0.035*	
C49	0.1639 (2)	0.36053 (19)	−0.07085 (10)	0.0280 (4)	
H49A	0.107004	0.407064	−0.100105	0.034*	
H49B	0.134997	0.285437	−0.076387	0.034*	
C50	0.06417 (18)	0.38286 (19)	0.01145 (10)	0.0261 (4)	
H50A	0.045586	0.305520	0.014122	0.031*	
H50B	0.001079	0.417187	−0.021125	0.031*	
C51	0.07717 (19)	0.43658 (18)	0.07439 (11)	0.0270 (4)	
H51A	0.002443	0.436712	0.085388	0.032*	
H51B	0.134125	0.397073	0.107800	0.032*	
C52	0.15078 (19)	0.6018 (2)	0.13300 (10)	0.0275 (4)	
H52	0.217019	0.560464	0.160068	0.033*	
C53	0.1913 (2)	0.7163 (2)	0.12757 (11)	0.0332 (5)	
H53A	0.130624	0.755035	0.095749	0.040*	
H53B	0.200141	0.752920	0.168719	0.040*	
C54	0.3029 (2)	0.7258 (2)	0.10930 (11)	0.0309 (5)	
H54A	0.329227	0.801741	0.113948	0.037*	
H54B	0.362415	0.681190	0.138227	0.037*	
C55	0.4260 (2)	0.4763 (2)	−0.12123 (12)	0.0355 (5)	
H55A	0.404487	0.441802	−0.162925	0.053*	
H55B	0.487589	0.434797	−0.092717	0.053*	
H55C	0.453063	0.549845	−0.125312	0.053*	
C56	0.0530 (2)	0.6009 (2)	0.16609 (12)	0.0381 (6)	
H56A	−0.015181	0.636318	0.139012	0.057*	
H56B	0.077832	0.639574	0.206404	0.057*	
H56C	0.034031	0.526216	0.173953	0.057*	
S1	0.6918 (7)	0.8003 (6)	0.4734 (3)	0.0332 (11)	0.50 (2)
O1	0.6705 (15)	0.7293 (12)	0.4194 (5)	0.099 (4)	0.50 (2)
O2	0.7769 (14)	0.8842 (10)	0.4875 (8)	0.049 (3)	0.50 (2)
O3	0.5941 (9)	0.8222 (11)	0.4981 (7)	0.045 (2)	0.50 (2)
F1	0.8656 (9)	0.6762 (12)	0.5194 (7)	0.054 (2)	0.50 (2)
F2	0.7082 (10)	0.6104 (7)	0.5296 (6)	0.054 (2)	0.50 (2)
F3	0.7786 (8)	0.7397 (10)	0.5895 (4)	0.062 (2)	0.50 (2)
C57	0.7676 (10)	0.7009 (9)	0.5326 (5)	0.037 (2)	0.50 (2)
S1B	0.7044 (6)	0.7879 (6)	0.4723 (3)	0.0309 (9)	0.50 (2)
O1B	0.7133 (8)	0.7290 (9)	0.4177 (4)	0.055 (2)	0.50 (2)
O2B	0.7640 (16)	0.8870 (12)	0.4692 (7)	0.050 (3)	0.50 (2)
O3B	0.5915 (9)	0.8073 (13)	0.4787 (5)	0.045 (2)	0.50 (2)
F1B	0.8866 (9)	0.6754 (12)	0.5320 (5)	0.0443 (17)	0.50 (2)
F2B	0.7322 (10)	0.6263 (10)	0.5491 (7)	0.067 (3)	0.50 (2)
F3B	0.8162 (13)	0.7693 (10)	0.5904 (5)	0.081 (3)	0.50 (2)
C57B	0.7849 (11)	0.7114 (10)	0.5378 (6)	0.036 (2)	0.50 (2)
S2	0.76108 (5)	0.56611 (5)	−0.01361 (3)	0.03150 (14)	
O4	0.7606 (2)	0.4696 (2)	−0.04905 (13)	0.0746 (9)	
O5	0.87335 (14)	0.61100 (16)	0.01408 (9)	0.0363 (4)	
O6	0.67605 (17)	0.6462 (2)	−0.04039 (13)	0.0700 (8)	
F4	0.78556 (16)	0.44510 (19)	0.08685 (11)	0.0708 (7)	
F5	0.61133 (13)	0.47501 (17)	0.03822 (9)	0.0522 (5)	
F6	0.7121 (2)	0.59882 (19)	0.09421 (10)	0.0754 (7)	
C58	0.7159 (2)	0.5187 (2)	0.05455 (12)	0.0333 (5)	
C59	0.4912 (6)	0.7261 (7)	0.2843 (4)	0.052 (2)	0.545 (12)
H59A	0.435121	0.784819	0.268751	0.062*	0.545 (12)
H59B	0.528091	0.739423	0.329702	0.062*	0.545 (12)
Cl1	0.4161 (7)	0.6001 (5)	0.2761 (3)	0.0557 (11)	0.545 (12)
Cl2	0.5971 (4)	0.7286 (3)	0.24236 (19)	0.0778 (8)	0.545 (12)
C59B	0.5033 (7)	0.6857 (7)	0.3089 (4)	0.0481 (19)	0.455 (12)
H59C	0.566395	0.643628	0.337059	0.058*	0.455 (12)
H59D	0.477247	0.740591	0.334912	0.058*	0.455 (12)
Cl1B	0.3905 (8)	0.6011 (7)	0.2748 (4)	0.0624 (17)	0.455 (12)
Cl2B	0.5550 (6)	0.7505 (3)	0.24951 (15)	0.0614 (13)	0.455 (12)

### (5,12-Dimethyl-1,4,8,11-tetraazacyclotetradecane)bis(phenylethynyl)cobalt(III) trifluoromethanesulfonate–dichloromethane (2/1) (2) Atomic displacement parameters (Å^2^)

**Table:**

	*U*^11^	*U*^22^	*U*^33^	*U*^12^	*U*^13^	*U*^23^
Co1	0.02035 (12)	0.01829 (12)	0.02225 (12)	−0.00115 (10)	0.01053 (9)	0.00103 (10)
N1	0.0258 (9)	0.0234 (9)	0.0276 (9)	−0.0002 (7)	0.0105 (7)	−0.0012 (7)
N2	0.0274 (8)	0.0278 (10)	0.0228 (8)	−0.0068 (7)	0.0069 (6)	0.0003 (7)
N3	0.0342 (10)	0.0217 (9)	0.0375 (10)	−0.0002 (8)	0.0191 (8)	−0.0024 (8)
N4	0.0220 (8)	0.0297 (10)	0.0312 (9)	−0.0030 (7)	0.0088 (7)	0.0039 (8)
C1	0.0213 (9)	0.0213 (10)	0.0255 (10)	−0.0017 (8)	0.0092 (7)	−0.0009 (8)
C2	0.0240 (9)	0.0225 (10)	0.0243 (9)	−0.0033 (8)	0.0082 (7)	−0.0031 (8)
C3	0.0239 (9)	0.0256 (10)	0.0181 (8)	−0.0094 (8)	0.0072 (7)	−0.0037 (7)
C4	0.0331 (11)	0.0298 (12)	0.0290 (11)	−0.0037 (9)	0.0142 (9)	−0.0035 (9)
C5	0.0426 (13)	0.0474 (14)	0.0305 (11)	−0.0165 (13)	0.0207 (10)	−0.0068 (12)
C6	0.0517 (16)	0.0579 (18)	0.0193 (10)	−0.0209 (14)	0.0100 (10)	0.0040 (11)
C7	0.0420 (14)	0.0449 (15)	0.0262 (11)	−0.0104 (12)	−0.0013 (10)	0.0122 (10)
C8	0.0240 (10)	0.0372 (13)	0.0276 (11)	−0.0039 (9)	0.0035 (8)	0.0030 (9)
C9	0.0262 (9)	0.0236 (10)	0.0265 (9)	−0.0025 (9)	0.0120 (7)	−0.0001 (9)
C10	0.0293 (10)	0.0253 (11)	0.0291 (10)	−0.0042 (9)	0.0129 (8)	−0.0017 (8)
C11	0.0312 (11)	0.0192 (10)	0.0290 (10)	−0.0017 (8)	0.0163 (8)	0.0007 (8)
C12	0.0299 (11)	0.0257 (11)	0.0342 (12)	0.0001 (9)	0.0110 (9)	0.0017 (9)
C13	0.0503 (15)	0.0301 (12)	0.0312 (12)	0.0072 (11)	0.0133 (11)	0.0035 (10)
C14	0.0528 (16)	0.0325 (14)	0.0447 (14)	0.0062 (11)	0.0312 (12)	0.0114 (11)
C15	0.0414 (14)	0.0362 (15)	0.0600 (18)	−0.0104 (11)	0.0266 (13)	0.0064 (12)
C16	0.0377 (13)	0.0325 (13)	0.0378 (13)	−0.0110 (10)	0.0137 (10)	0.0005 (10)
C17	0.0261 (10)	0.0316 (12)	0.0326 (11)	0.0062 (9)	0.0094 (8)	0.0001 (9)
C18	0.0244 (10)	0.0348 (13)	0.0300 (11)	0.0020 (9)	0.0060 (8)	0.0050 (9)
C19	0.0404 (13)	0.0349 (13)	0.0265 (11)	−0.0136 (10)	0.0075 (9)	−0.0046 (9)
C20	0.0594 (16)	0.0325 (14)	0.0382 (12)	−0.0123 (12)	0.0159 (12)	−0.0104 (11)
C21	0.0562 (16)	0.0296 (13)	0.0418 (13)	−0.0018 (11)	0.0267 (12)	−0.0115 (11)
C22	0.0313 (12)	0.0285 (12)	0.0592 (16)	0.0079 (10)	0.0253 (11)	0.0015 (11)
C23	0.0238 (11)	0.0369 (13)	0.0486 (14)	0.0072 (10)	0.0138 (10)	0.0115 (11)
C24	0.0277 (10)	0.0395 (14)	0.0285 (10)	−0.0078 (10)	0.0034 (8)	0.0002 (10)
C25	0.0382 (13)	0.0348 (13)	0.0358 (12)	−0.0114 (10)	0.0084 (10)	−0.0114 (10)
C26	0.0375 (12)	0.0271 (11)	0.0315 (11)	−0.0008 (10)	0.0121 (9)	−0.0078 (9)
C27	0.0493 (16)	0.0542 (18)	0.0362 (13)	−0.0227 (14)	0.0008 (11)	−0.0007 (12)
C28	0.0317 (13)	0.064 (2)	0.0458 (15)	−0.0097 (13)	−0.0022 (11)	0.0031 (14)
Co2	0.01427 (11)	0.01756 (12)	0.01917 (12)	0.00037 (10)	0.00138 (8)	0.00328 (10)
N5	0.0237 (8)	0.0208 (9)	0.0270 (9)	−0.0001 (7)	0.0036 (7)	0.0014 (7)
N6	0.0191 (7)	0.0285 (9)	0.0245 (8)	0.0020 (8)	0.0064 (6)	0.0021 (8)
N7	0.0204 (8)	0.0229 (9)	0.0229 (8)	−0.0014 (7)	0.0041 (6)	0.0010 (7)
N8	0.0204 (7)	0.0219 (8)	0.0218 (7)	0.0011 (7)	0.0043 (6)	0.0024 (7)
C29	0.0194 (9)	0.0221 (10)	0.0227 (9)	0.0012 (7)	0.0031 (7)	0.0023 (7)
C30	0.0219 (9)	0.0258 (10)	0.0221 (9)	0.0018 (8)	0.0043 (7)	0.0032 (8)
C31	0.0182 (9)	0.0293 (11)	0.0187 (9)	0.0040 (8)	0.0043 (7)	0.0049 (8)
C32	0.0262 (10)	0.0293 (12)	0.0292 (10)	0.0030 (8)	0.0053 (8)	0.0041 (8)
C33	0.0366 (12)	0.0335 (13)	0.0346 (11)	0.0114 (10)	0.0098 (9)	0.0139 (10)
C34	0.0259 (10)	0.0513 (15)	0.0263 (10)	0.0128 (11)	0.0034 (8)	0.0145 (11)
C35	0.0286 (12)	0.0498 (16)	0.0339 (12)	0.0005 (11)	−0.0040 (9)	0.0050 (11)
C36	0.0280 (11)	0.0312 (12)	0.0329 (11)	−0.0007 (9)	−0.0019 (9)	0.0053 (9)
C37	0.0175 (8)	0.0216 (9)	0.0231 (9)	−0.0011 (7)	0.0050 (7)	0.0021 (7)
C38	0.0211 (9)	0.0221 (10)	0.0214 (9)	0.0025 (8)	0.0041 (7)	0.0013 (7)
C39	0.0241 (9)	0.0193 (9)	0.0179 (8)	0.0034 (7)	0.0012 (7)	−0.0007 (7)
C40	0.0261 (10)	0.0272 (10)	0.0255 (10)	−0.0014 (8)	0.0002 (8)	0.0021 (8)
C41	0.0279 (11)	0.0369 (13)	0.0352 (12)	0.0005 (10)	−0.0071 (9)	0.0039 (10)
C42	0.0452 (13)	0.0383 (14)	0.0256 (10)	0.0069 (11)	−0.0061 (9)	0.0118 (10)
C43	0.0466 (13)	0.0333 (13)	0.0255 (10)	0.0019 (11)	0.0067 (9)	0.0109 (9)
C44	0.0279 (10)	0.0285 (11)	0.0253 (10)	0.0038 (8)	0.0060 (8)	0.0042 (8)
C45	0.0237 (10)	0.0274 (11)	0.0406 (12)	−0.0075 (9)	0.0081 (9)	−0.0018 (9)
C46	0.0246 (10)	0.0291 (12)	0.0388 (12)	−0.0056 (9)	0.0116 (9)	0.0025 (10)
C47	0.0265 (10)	0.0318 (12)	0.0228 (10)	−0.0004 (9)	0.0068 (8)	0.0008 (8)
C48	0.0331 (11)	0.0280 (12)	0.0293 (11)	−0.0006 (9)	0.0118 (9)	−0.0057 (9)
C49	0.0306 (11)	0.0264 (11)	0.0251 (10)	−0.0036 (9)	0.0042 (8)	−0.0038 (8)
C50	0.0231 (10)	0.0252 (11)	0.0305 (11)	−0.0067 (8)	0.0080 (8)	0.0007 (8)
C51	0.0263 (10)	0.0263 (11)	0.0307 (11)	−0.0011 (8)	0.0115 (8)	0.0042 (9)
C52	0.0288 (10)	0.0313 (12)	0.0220 (9)	0.0011 (9)	0.0060 (8)	−0.0026 (8)
C53	0.0380 (12)	0.0321 (13)	0.0297 (11)	0.0016 (10)	0.0096 (9)	−0.0045 (9)
C54	0.0345 (11)	0.0258 (12)	0.0309 (10)	−0.0042 (9)	0.0063 (9)	−0.0055 (9)
C55	0.0338 (12)	0.0413 (14)	0.0357 (12)	0.0018 (11)	0.0169 (10)	−0.0022 (11)
C56	0.0421 (13)	0.0438 (15)	0.0322 (12)	0.0016 (12)	0.0165 (10)	−0.0013 (11)
S1	0.040 (2)	0.0276 (14)	0.0287 (12)	0.0064 (12)	0.0040 (11)	−0.0018 (8)
O1	0.113 (8)	0.107 (6)	0.051 (4)	0.049 (6)	−0.025 (5)	−0.042 (4)
O2	0.046 (3)	0.022 (3)	0.089 (8)	−0.006 (3)	0.038 (5)	−0.005 (4)
O3	0.022 (2)	0.039 (3)	0.076 (7)	0.000 (2)	0.020 (4)	0.007 (4)
F1	0.018 (3)	0.057 (3)	0.082 (6)	0.018 (3)	0.000 (3)	−0.012 (4)
F2	0.053 (4)	0.028 (2)	0.094 (6)	0.006 (2)	0.042 (4)	0.005 (3)
F3	0.060 (4)	0.096 (6)	0.029 (3)	0.021 (3)	0.010 (3)	−0.007 (3)
C57	0.024 (3)	0.044 (4)	0.044 (4)	0.009 (3)	0.012 (3)	−0.012 (3)
S1B	0.0232 (11)	0.0262 (17)	0.0450 (15)	−0.0017 (13)	0.0119 (8)	−0.0008 (10)
O1B	0.058 (4)	0.074 (4)	0.033 (3)	0.003 (4)	0.009 (3)	−0.010 (3)
O2B	0.064 (6)	0.035 (3)	0.068 (6)	−0.002 (3)	0.047 (5)	0.010 (4)
O3B	0.025 (2)	0.058 (5)	0.051 (5)	0.006 (3)	0.011 (3)	0.011 (4)
F1B	0.013 (3)	0.065 (3)	0.051 (3)	0.012 (3)	0.003 (3)	0.004 (3)
F2B	0.053 (4)	0.060 (5)	0.091 (6)	0.008 (3)	0.026 (4)	0.042 (4)
F3B	0.095 (7)	0.077 (6)	0.046 (3)	0.045 (5)	−0.025 (4)	−0.024 (3)
C57B	0.027 (4)	0.034 (3)	0.044 (4)	−0.003 (3)	0.004 (3)	0.000 (3)
S2	0.0218 (2)	0.0462 (4)	0.0262 (3)	−0.0002 (2)	0.00569 (19)	0.0004 (2)
O4	0.0803 (18)	0.0837 (19)	0.0732 (17)	−0.0383 (16)	0.0435 (14)	−0.0486 (15)
O5	0.0229 (8)	0.0410 (10)	0.0455 (10)	−0.0031 (7)	0.0101 (7)	0.0012 (8)
O6	0.0286 (10)	0.100 (2)	0.0794 (16)	0.0186 (12)	0.0114 (10)	0.0540 (16)
F4	0.0403 (10)	0.0843 (15)	0.0891 (15)	0.0137 (10)	0.0192 (9)	0.0559 (13)
F5	0.0273 (8)	0.0698 (12)	0.0620 (11)	−0.0072 (8)	0.0164 (7)	0.0113 (10)
F6	0.1158 (18)	0.0679 (14)	0.0638 (12)	−0.0239 (13)	0.0612 (13)	−0.0208 (11)
C58	0.0242 (10)	0.0376 (14)	0.0381 (12)	0.0006 (9)	0.0080 (9)	0.0021 (10)
C59	0.050 (3)	0.050 (4)	0.050 (4)	0.008 (3)	0.003 (3)	−0.019 (3)
Cl1	0.057 (3)	0.0634 (18)	0.0486 (14)	0.0088 (15)	0.0164 (14)	0.0098 (12)
Cl2	0.0591 (16)	0.0467 (13)	0.135 (2)	−0.0116 (11)	0.0386 (14)	−0.0176 (13)
C59B	0.060 (4)	0.037 (4)	0.037 (4)	0.008 (3)	−0.003 (3)	0.001 (3)
Cl1B	0.060 (3)	0.091 (3)	0.0347 (14)	−0.015 (2)	0.0102 (16)	−0.0079 (15)
Cl2B	0.067 (2)	0.0463 (14)	0.0701 (15)	−0.0101 (15)	0.0165 (13)	−0.0004 (10)

### (5,12-Dimethyl-1,4,8,11-tetraazacyclotetradecane)bis(phenylethynyl)cobalt(III) trifluoromethanesulfonate–dichloromethane (2/1) (2) Geometric parameters (Å, º)

**Table:**

Co1—C9	1.926 (2)	N8—C51	1.482 (3)
Co1—C1	1.927 (2)	N8—C52	1.491 (3)
Co1—N1	1.9768 (19)	N8—H8N	1.0000
Co1—N3	1.982 (2)	C29—C30	1.214 (3)
Co1—N4	1.9985 (18)	C30—C31	1.441 (3)
Co1—N2	2.0126 (18)	C31—C32	1.386 (3)
N1—C26	1.473 (3)	C31—C36	1.399 (3)
N1—C17	1.477 (3)	C32—C33	1.395 (3)
N1—H1N	1.0000	C32—H32	0.9500
N2—C18	1.487 (3)	C33—C34	1.374 (4)
N2—C19	1.494 (3)	C33—H33	0.9500
N2—H2N	1.0000	C34—C35	1.386 (4)
N3—C21	1.472 (3)	C34—H34	0.9500
N3—C22	1.479 (3)	C35—C36	1.392 (3)
N3—H3N	1.0000	C35—H35	0.9500
N4—C23	1.478 (3)	C36—H36	0.9500
N4—C24	1.503 (3)	C37—C38	1.212 (3)
N4—H4N	1.0000	C38—C39	1.440 (3)
C1—C2	1.215 (3)	C39—C44	1.396 (3)
C2—C3	1.438 (3)	C39—C40	1.402 (3)
C3—C8	1.396 (3)	C40—C41	1.394 (3)
C3—C4	1.401 (3)	C40—H40	0.9500
C4—C5	1.383 (3)	C41—C42	1.388 (4)
C4—H4	0.9500	C41—H41	0.9500
C5—C6	1.373 (4)	C42—C43	1.383 (4)
C5—H5	0.9500	C42—H42	0.9500
C6—C7	1.375 (4)	C43—C44	1.390 (3)
C6—H6	0.9500	C43—H43	0.9500
C7—C8	1.395 (3)	C44—H44	0.9500
C7—H7	0.9500	C45—C46	1.498 (3)
C8—H8	0.9500	C45—H45A	0.9900
C9—C10	1.206 (3)	C45—H45B	0.9900
C10—C11	1.435 (3)	C46—H46A	0.9900
C11—C16	1.391 (3)	C46—H46B	0.9900
C11—C12	1.409 (3)	C47—C48	1.517 (3)
C12—C13	1.376 (4)	C47—C55	1.528 (3)
C12—H12	0.9500	C47—H47	1.0000
C13—C14	1.381 (4)	C48—C49	1.521 (3)
C13—H13	0.9500	C48—H48A	0.9900
C14—C15	1.383 (4)	C48—H48B	0.9900
C14—H14	0.9500	C49—H49A	0.9900
C15—C16	1.377 (4)	C49—H49B	0.9900
C15—H15	0.9500	C50—C51	1.502 (3)
C16—H16	0.9500	C50—H50A	0.9900
C17—C18	1.509 (3)	C50—H50B	0.9900
C17—H17A	0.9900	C51—H51A	0.9900
C17—H17B	0.9900	C51—H51B	0.9900
C18—H18A	0.9900	C52—C53	1.515 (3)
C18—H18B	0.9900	C52—C56	1.536 (3)
C19—C20	1.518 (4)	C52—H52	1.0000
C19—C27	1.523 (4)	C53—C54	1.504 (3)
C19—H19	1.0000	C53—H53A	0.9900
C20—C21	1.507 (4)	C53—H53B	0.9900
C20—H20A	0.9900	C54—H54A	0.9900
C20—H20B	0.9900	C54—H54B	0.9900
C21—H21A	0.9900	C55—H55A	0.9800
C21—H21B	0.9900	C55—H55B	0.9800
C22—C23	1.485 (4)	C55—H55C	0.9800
C22—H22A	0.9900	C56—H56A	0.9800
C22—H22B	0.9900	C56—H56B	0.9800
C23—H23A	0.9900	C56—H56C	0.9800
C23—H23B	0.9900	S1—O2	1.435 (11)
C24—C25	1.511 (4)	S1—O1	1.442 (10)
C24—C28	1.535 (3)	S1—O3	1.443 (10)
C24—H24	1.0000	S1—C57	1.845 (11)
C25—C26	1.517 (4)	F1—C57	1.322 (11)
C25—H25A	0.9900	F2—C57	1.323 (12)
C25—H25B	0.9900	F3—C57	1.310 (11)
C26—H26A	0.9900	S1B—O3B	1.422 (10)
C26—H26B	0.9900	S1B—O1B	1.430 (10)
C27—H27A	0.9800	S1B—O2B	1.433 (12)
C27—H27B	0.9800	S1B—C57B	1.779 (10)
C27—H27C	0.9800	F1B—C57B	1.339 (12)
C28—H28A	0.9800	F2B—C57B	1.287 (12)
C28—H28B	0.9800	F3B—C57B	1.325 (12)
C28—H28C	0.9800	S2—O4	1.425 (3)
Co2—C37	1.9262 (19)	S2—O6	1.435 (2)
Co2—C29	1.9273 (19)	S2—O5	1.4380 (17)
Co2—N7	1.9789 (18)	S2—C58	1.818 (3)
Co2—N5	1.9835 (18)	F4—C58	1.312 (3)
Co2—N6	2.0067 (17)	F5—C58	1.328 (3)
Co2—N8	2.0071 (16)	F6—C58	1.329 (3)
N5—C54	1.478 (3)	C59—Cl2	1.757 (7)
N5—C45	1.480 (3)	C59—Cl1	1.789 (9)
N5—H5N	1.0000	C59—H59A	0.9900
N6—C46	1.478 (3)	C59—H59B	0.9900
N6—C47	1.501 (3)	C59B—Cl1B	1.722 (10)
N6—H6N	1.0000	C59B—Cl2B	1.777 (9)
N7—C49	1.470 (3)	C59B—H59C	0.9900
N7—C50	1.480 (3)	C59B—H59D	0.9900
N7—H7N	1.0000		
			
C9—Co1—C1	179.67 (9)	C46—N6—H6N	105.7
C9—Co1—N1	87.08 (9)	C47—N6—H6N	105.7
C1—Co1—N1	92.91 (8)	Co2—N6—H6N	105.7
C9—Co1—N3	91.84 (9)	C49—N7—C50	111.01 (17)
C1—Co1—N3	88.17 (9)	C49—N7—Co2	117.62 (14)
N1—Co1—N3	178.87 (8)	C50—N7—Co2	107.65 (14)
C9—Co1—N4	89.79 (8)	C49—N7—H7N	106.7
C1—Co1—N4	90.54 (8)	C50—N7—H7N	106.7
N1—Co1—N4	93.92 (8)	Co2—N7—H7N	106.7
N3—Co1—N4	86.43 (8)	C51—N8—C52	111.41 (16)
C9—Co1—N2	90.20 (8)	C51—N8—Co2	106.60 (13)
C1—Co1—N2	89.47 (8)	C52—N8—Co2	118.33 (13)
N1—Co1—N2	86.35 (8)	C51—N8—H8N	106.6
N3—Co1—N2	93.30 (8)	C52—N8—H8N	106.6
N4—Co1—N2	179.73 (9)	Co2—N8—H8N	106.6
C26—N1—C17	110.60 (18)	C30—C29—Co2	171.40 (19)
C26—N1—Co1	119.21 (15)	C29—C30—C31	177.2 (2)
C17—N1—Co1	107.90 (14)	C32—C31—C36	118.65 (19)
C26—N1—H1N	106.1	C32—C31—C30	121.6 (2)
C17—N1—H1N	106.1	C36—C31—C30	119.7 (2)
Co1—N1—H1N	106.1	C31—C32—C33	120.3 (2)
C18—N2—C19	111.72 (18)	C31—C32—H32	119.9
C18—N2—Co1	107.15 (13)	C33—C32—H32	119.9
C19—N2—Co1	119.05 (15)	C34—C33—C32	121.0 (2)
C18—N2—H2N	106.0	C34—C33—H33	119.5
C19—N2—H2N	106.0	C32—C33—H33	119.5
Co1—N2—H2N	106.0	C33—C34—C35	119.3 (2)
C21—N3—C22	111.53 (19)	C33—C34—H34	120.4
C21—N3—Co1	118.69 (16)	C35—C34—H34	120.4
C22—N3—Co1	106.67 (15)	C34—C35—C36	120.3 (2)
C21—N3—H3N	106.4	C34—C35—H35	119.8
C22—N3—H3N	106.4	C36—C35—H35	119.8
Co1—N3—H3N	106.4	C35—C36—C31	120.5 (2)
C23—N4—C24	112.04 (19)	C35—C36—H36	119.8
C23—N4—Co1	106.26 (15)	C31—C36—H36	119.8
C24—N4—Co1	117.80 (14)	C38—C37—Co2	174.23 (19)
C23—N4—H4N	106.7	C37—C38—C39	177.7 (2)
C24—N4—H4N	106.7	C44—C39—C40	118.59 (18)
Co1—N4—H4N	106.7	C44—C39—C38	120.33 (19)
C2—C1—Co1	174.06 (19)	C40—C39—C38	121.08 (19)
C1—C2—C3	178.3 (2)	C41—C40—C39	120.6 (2)
C8—C3—C4	118.5 (2)	C41—C40—H40	119.7
C8—C3—C2	120.8 (2)	C39—C40—H40	119.7
C4—C3—C2	120.7 (2)	C42—C41—C40	119.9 (2)
C5—C4—C3	120.7 (2)	C42—C41—H41	120.1
C5—C4—H4	119.7	C40—C41—H41	120.1
C3—C4—H4	119.7	C43—C42—C41	120.1 (2)
C6—C5—C4	120.2 (2)	C43—C42—H42	120.0
C6—C5—H5	119.9	C41—C42—H42	120.0
C4—C5—H5	119.9	C42—C43—C44	120.3 (2)
C5—C6—C7	120.2 (2)	C42—C43—H43	119.9
C5—C6—H6	119.9	C44—C43—H43	119.9
C7—C6—H6	119.9	C43—C44—C39	120.6 (2)
C6—C7—C8	120.5 (3)	C43—C44—H44	119.7
C6—C7—H7	119.8	C39—C44—H44	119.7
C8—C7—H7	119.8	N5—C45—C46	108.36 (18)
C7—C8—C3	119.9 (2)	N5—C45—H45A	110.0
C7—C8—H8	120.0	C46—C45—H45A	110.0
C3—C8—H8	120.0	N5—C45—H45B	110.0
C10—C9—Co1	173.7 (2)	C46—C45—H45B	110.0
C9—C10—C11	177.6 (2)	H45A—C45—H45B	108.4
C16—C11—C12	117.9 (2)	N6—C46—C45	107.75 (19)
C16—C11—C10	121.5 (2)	N6—C46—H46A	110.2
C12—C11—C10	120.5 (2)	C45—C46—H46A	110.2
C13—C12—C11	120.6 (2)	N6—C46—H46B	110.2
C13—C12—H12	119.7	C45—C46—H46B	110.2
C11—C12—H12	119.7	H46A—C46—H46B	108.5
C12—C13—C14	120.5 (2)	N6—C47—C48	110.43 (18)
C12—C13—H13	119.8	N6—C47—C55	110.81 (19)
C14—C13—H13	119.8	C48—C47—C55	109.9 (2)
C13—C14—C15	119.7 (2)	N6—C47—H47	108.5
C13—C14—H14	120.2	C48—C47—H47	108.5
C15—C14—H14	120.2	C55—C47—H47	108.5
C16—C15—C14	120.3 (3)	C47—C48—C49	115.0 (2)
C16—C15—H15	119.9	C47—C48—H48A	108.5
C14—C15—H15	119.9	C49—C48—H48A	108.5
C15—C16—C11	121.1 (3)	C47—C48—H48B	108.5
C15—C16—H16	119.4	C49—C48—H48B	108.5
C11—C16—H16	119.4	H48A—C48—H48B	107.5
N1—C17—C18	108.18 (18)	N7—C49—C48	111.35 (18)
N1—C17—H17A	110.1	N7—C49—H49A	109.4
C18—C17—H17A	110.1	C48—C49—H49A	109.4
N1—C17—H17B	110.1	N7—C49—H49B	109.4
C18—C17—H17B	110.1	C48—C49—H49B	109.4
H17A—C17—H17B	108.4	H49A—C49—H49B	108.0
N2—C18—C17	107.98 (18)	N7—C50—C51	107.53 (17)
N2—C18—H18A	110.1	N7—C50—H50A	110.2
C17—C18—H18A	110.1	C51—C50—H50A	110.2
N2—C18—H18B	110.1	N7—C50—H50B	110.2
C17—C18—H18B	110.1	C51—C50—H50B	110.2
H18A—C18—H18B	108.4	H50A—C50—H50B	108.5
N2—C19—C20	110.3 (2)	N8—C51—C50	108.08 (17)
N2—C19—C27	112.1 (2)	N8—C51—H51A	110.1
C20—C19—C27	110.9 (2)	C50—C51—H51A	110.1
N2—C19—H19	107.8	N8—C51—H51B	110.1
C20—C19—H19	107.8	C50—C51—H51B	110.1
C27—C19—H19	107.8	H51A—C51—H51B	108.4
C21—C20—C19	115.2 (2)	N8—C52—C53	110.84 (18)
C21—C20—H20A	108.5	N8—C52—C56	112.26 (19)
C19—C20—H20A	108.5	C53—C52—C56	110.3 (2)
C21—C20—H20B	108.5	N8—C52—H52	107.8
C19—C20—H20B	108.5	C53—C52—H52	107.8
H20A—C20—H20B	107.5	C56—C52—H52	107.8
N3—C21—C20	111.8 (2)	C54—C53—C52	115.0 (2)
N3—C21—H21A	109.3	C54—C53—H53A	108.5
C20—C21—H21A	109.3	C52—C53—H53A	108.5
N3—C21—H21B	109.3	C54—C53—H53B	108.5
C20—C21—H21B	109.3	C52—C53—H53B	108.5
H21A—C21—H21B	107.9	H53A—C53—H53B	107.5
N3—C22—C23	107.92 (19)	N5—C54—C53	111.57 (18)
N3—C22—H22A	110.1	N5—C54—H54A	109.3
C23—C22—H22A	110.1	C53—C54—H54A	109.3
N3—C22—H22B	110.1	N5—C54—H54B	109.3
C23—C22—H22B	110.1	C53—C54—H54B	109.3
H22A—C22—H22B	108.4	H54A—C54—H54B	108.0
N4—C23—C22	107.6 (2)	C47—C55—H55A	109.5
N4—C23—H23A	110.2	C47—C55—H55B	109.5
C22—C23—H23A	110.2	H55A—C55—H55B	109.5
N4—C23—H23B	110.2	C47—C55—H55C	109.5
C22—C23—H23B	110.2	H55A—C55—H55C	109.5
H23A—C23—H23B	108.5	H55B—C55—H55C	109.5
N4—C24—C25	110.03 (19)	C52—C56—H56A	109.5
N4—C24—C28	111.9 (2)	C52—C56—H56B	109.5
C25—C24—C28	110.9 (2)	H56A—C56—H56B	109.5
N4—C24—H24	108.0	C52—C56—H56C	109.5
C25—C24—H24	108.0	H56A—C56—H56C	109.5
C28—C24—H24	108.0	H56B—C56—H56C	109.5
C24—C25—C26	114.4 (2)	O2—S1—O1	125.5 (12)
C24—C25—H25A	108.7	O2—S1—O3	112.9 (9)
C26—C25—H25A	108.7	O1—S1—O3	115.9 (7)
C24—C25—H25B	108.7	O2—S1—C57	98.2 (8)
C26—C25—H25B	108.7	O1—S1—C57	96.7 (8)
H25A—C25—H25B	107.6	O3—S1—C57	99.4 (7)
N1—C26—C25	112.5 (2)	F3—C57—F1	115.0 (10)
N1—C26—H26A	109.1	F3—C57—F2	106.0 (9)
C25—C26—H26A	109.1	F1—C57—F2	107.1 (10)
N1—C26—H26B	109.1	F3—C57—S1	109.3 (8)
C25—C26—H26B	109.1	F1—C57—S1	107.7 (9)
H26A—C26—H26B	107.8	F2—C57—S1	111.8 (8)
C19—C27—H27A	109.5	O3B—S1B—O1B	117.0 (6)
C19—C27—H27B	109.5	O3B—S1B—O2B	111.2 (11)
H27A—C27—H27B	109.5	O1B—S1B—O2B	104.5 (9)
C19—C27—H27C	109.5	O3B—S1B—C57B	109.9 (8)
H27A—C27—H27C	109.5	O1B—S1B—C57B	105.0 (7)
H27B—C27—H27C	109.5	O2B—S1B—C57B	108.8 (8)
C24—C28—H28A	109.5	F2B—C57B—F3B	108.5 (10)
C24—C28—H28B	109.5	F2B—C57B—F1B	104.7 (11)
H28A—C28—H28B	109.5	F3B—C57B—F1B	102.0 (10)
C24—C28—H28C	109.5	F2B—C57B—S1B	113.7 (8)
H28A—C28—H28C	109.5	F3B—C57B—S1B	112.4 (9)
H28B—C28—H28C	109.5	F1B—C57B—S1B	114.6 (9)
C37—Co2—C29	177.67 (9)	O4—S2—O6	117.27 (19)
C37—Co2—N7	92.41 (8)	O4—S2—O5	115.03 (14)
C29—Co2—N7	88.10 (8)	O6—S2—O5	113.12 (15)
C37—Co2—N5	87.84 (8)	O4—S2—C58	102.53 (14)
C29—Co2—N5	91.66 (8)	O6—S2—C58	102.98 (12)
N7—Co2—N5	179.53 (8)	O5—S2—C58	103.30 (11)
C37—Co2—N6	90.34 (8)	F4—C58—F5	106.6 (2)
C29—Co2—N6	87.36 (8)	F4—C58—F6	107.1 (2)
N7—Co2—N6	94.17 (8)	F5—C58—F6	106.7 (2)
N5—Co2—N6	86.23 (8)	F4—C58—S2	112.40 (18)
C37—Co2—N8	89.93 (8)	F5—C58—S2	112.21 (17)
C29—Co2—N8	92.37 (8)	F6—C58—S2	111.39 (18)
N7—Co2—N8	86.38 (7)	Cl2—C59—Cl1	112.2 (4)
N5—Co2—N8	93.23 (8)	Cl2—C59—H59A	109.2
N6—Co2—N8	179.38 (8)	Cl1—C59—H59A	109.2
C54—N5—C45	110.68 (17)	Cl2—C59—H59B	109.2
C54—N5—Co2	118.69 (14)	Cl1—C59—H59B	109.2
C45—N5—Co2	107.45 (14)	H59A—C59—H59B	107.9
C54—N5—H5N	106.4	Cl1B—C59B—Cl2B	110.2 (5)
C45—N5—H5N	106.4	Cl1B—C59B—H59C	109.6
Co2—N5—H5N	106.4	Cl2B—C59B—H59C	109.6
C46—N6—C47	112.16 (17)	Cl1B—C59B—H59D	109.6
C46—N6—Co2	106.98 (14)	Cl2B—C59B—H59D	109.6
C47—N6—Co2	119.56 (13)	H59C—C59B—H59D	108.1
			
C8—C3—C4—C5	−0.8 (3)	C40—C39—C44—C43	−0.4 (3)
C2—C3—C4—C5	−179.7 (2)	C38—C39—C44—C43	179.2 (2)
C3—C4—C5—C6	0.8 (4)	C54—N5—C45—C46	170.65 (19)
C4—C5—C6—C7	0.0 (4)	Co2—N5—C45—C46	39.6 (2)
C5—C6—C7—C8	−0.8 (4)	C47—N6—C46—C45	173.08 (18)
C6—C7—C8—C3	0.8 (4)	Co2—N6—C46—C45	40.1 (2)
C4—C3—C8—C7	0.0 (3)	N5—C45—C46—N6	−53.6 (2)
C2—C3—C8—C7	179.0 (2)	C46—N6—C47—C48	−178.48 (18)
C16—C11—C12—C13	−0.4 (3)	Co2—N6—C47—C48	−52.1 (2)
C10—C11—C12—C13	−178.6 (2)	C46—N6—C47—C55	59.5 (2)
C11—C12—C13—C14	−0.5 (4)	Co2—N6—C47—C55	−174.14 (16)
C12—C13—C14—C15	0.7 (4)	N6—C47—C48—C49	66.6 (2)
C13—C14—C15—C16	−0.1 (4)	C55—C47—C48—C49	−170.80 (19)
C14—C15—C16—C11	−0.8 (4)	C50—N7—C49—C48	−176.88 (19)
C12—C11—C16—C15	1.1 (4)	Co2—N7—C49—C48	58.5 (2)
C10—C11—C16—C15	179.2 (2)	C47—C48—C49—N7	−71.2 (3)
C26—N1—C17—C18	−172.65 (19)	C49—N7—C50—C51	−170.66 (18)
Co1—N1—C17—C18	−40.6 (2)	Co2—N7—C50—C51	−40.62 (19)
C19—N2—C18—C17	−169.88 (19)	C52—N8—C51—C50	−170.37 (17)
Co1—N2—C18—C17	−37.8 (2)	Co2—N8—C51—C50	−39.93 (19)
N1—C17—C18—N2	52.5 (2)	N7—C50—C51—N8	54.1 (2)
C18—N2—C19—C20	−179.61 (19)	C51—N8—C52—C53	179.86 (18)
Co1—N2—C19—C20	54.6 (2)	Co2—N8—C52—C53	55.8 (2)
C18—N2—C19—C27	−55.5 (3)	C51—N8—C52—C56	−56.4 (2)
Co1—N2—C19—C27	178.71 (17)	Co2—N8—C52—C56	179.60 (16)
N2—C19—C20—C21	−67.5 (3)	N8—C52—C53—C54	−68.0 (2)
C27—C19—C20—C21	167.7 (2)	C56—C52—C53—C54	167.1 (2)
C22—N3—C21—C20	178.7 (2)	C45—N5—C54—C53	178.41 (19)
Co1—N3—C21—C20	−56.7 (3)	Co2—N5—C54—C53	−56.7 (2)
C19—C20—C21—N3	69.2 (3)	C52—C53—C54—N5	68.5 (3)
C21—N3—C22—C23	172.2 (2)	O2—S1—C57—F3	64.4 (11)
Co1—N3—C22—C23	41.1 (2)	O1—S1—C57—F3	−168.2 (12)
C24—N4—C23—C22	171.47 (19)	O3—S1—C57—F3	−50.5 (10)
Co1—N4—C23—C22	41.5 (2)	O2—S1—C57—F1	−61.2 (11)
N3—C22—C23—N4	−55.8 (3)	O1—S1—C57—F1	66.2 (12)
C23—N4—C24—C25	178.40 (19)	O3—S1—C57—F1	−176.1 (10)
Co1—N4—C24—C25	−57.9 (2)	O2—S1—C57—F2	−178.5 (10)
C23—N4—C24—C28	54.7 (3)	O1—S1—C57—F2	−51.1 (11)
Co1—N4—C24—C28	178.40 (18)	O3—S1—C57—F2	66.5 (10)
N4—C24—C25—C26	69.5 (3)	O3B—S1B—C57B—F2B	50.5 (14)
C28—C24—C25—C26	−166.2 (2)	O1B—S1B—C57B—F2B	−76.2 (13)
C17—N1—C26—C25	179.6 (2)	O2B—S1B—C57B—F2B	172.5 (13)
Co1—N1—C26—C25	53.7 (3)	O3B—S1B—C57B—F3B	−73.4 (13)
C24—C25—C26—N1	−67.7 (3)	O1B—S1B—C57B—F3B	160.0 (11)
C36—C31—C32—C33	−0.5 (3)	O2B—S1B—C57B—F3B	48.7 (14)
C30—C31—C32—C33	178.9 (2)	O3B—S1B—C57B—F1B	170.8 (11)
C31—C32—C33—C34	0.4 (4)	O1B—S1B—C57B—F1B	44.2 (12)
C32—C33—C34—C35	−0.2 (4)	O2B—S1B—C57B—F1B	−67.2 (14)
C33—C34—C35—C36	0.1 (4)	O4—S2—C58—F4	59.2 (3)
C34—C35—C36—C31	−0.2 (4)	O6—S2—C58—F4	−178.6 (2)
C32—C31—C36—C35	0.4 (4)	O5—S2—C58—F4	−60.7 (2)
C30—C31—C36—C35	−178.9 (2)	O4—S2—C58—F5	−61.0 (2)
C44—C39—C40—C41	0.0 (3)	O6—S2—C58—F5	61.2 (2)
C38—C39—C40—C41	−179.6 (2)	O5—S2—C58—F5	179.16 (19)
C39—C40—C41—C42	0.4 (4)	O4—S2—C58—F6	179.4 (2)
C40—C41—C42—C43	−0.3 (4)	O6—S2—C58—F6	−58.4 (2)
C41—C42—C43—C44	−0.1 (4)	O5—S2—C58—F6	59.6 (2)
C42—C43—C44—C39	0.5 (4)		

### (5,12-Dimethyl-1,4,8,11-tetraazacyclotetradecane)bis(phenylethynyl)cobalt(III) trifluoromethanesulfonate–dichloromethane (2/1) (2) Hydrogen-bond geometry (Å, º)

**Table:**

*D*—H···*A*	*D*—H	H···*A*	*D*···*A*	*D*—H···*A*
N1—H1*N*···F2^i^	1.00	2.43	3.298 (9)	145
N1—H1*N*···F2*B*^i^	1.00	2.59	3.504 (14)	151
N2—H2*N*···F1^ii^	1.00	2.61	3.482 (13)	146
N2—H2*N*···F1*B*^ii^	1.00	2.61	3.491 (11)	148
N3—H3*N*···O2^iii^	1.00	2.06	2.947 (15)	147
N3—H3*N*···O2*B*^iii^	1.00	2.13	3.053 (18)	153
N4—H4*N*···O3	1.00	2.14	3.001 (11)	143
N4—H4*N*···O3*B*	1.00	2.31	3.183 (12)	145
C21—H21*B*···Cl1*B*	0.99	2.94	3.779 (9)	144
C22—H22*B*···O3^iii^	0.99	2.49	3.401 (14)	152
C23—H23*B*···F2	0.99	2.59	3.419 (11)	142
C23—H23*B*···F2*B*	0.99	2.64	3.543 (12)	152
N5—H5*N*···O4^iv^	1.00	2.92	3.523 (4)	119
N6—H6*N*···F5	1.00	2.29	3.211 (2)	153
N7—H7*N*···O6^v^	1.00	2.69	3.575 (4)	148
N8—H8*N*···O5^ii^	1.00	2.05	2.960 (2)	150
C46—H46*B*···O6	0.99	2.52	3.483 (3)	163
C49—H49*B*···O5^v^	0.99	2.57	3.408 (3)	142
C51—H51*A*···F4^ii^	0.99	2.62	3.590 (3)	167
C52—H52···Cl1*B*	1.00	2.86	3.637 (8)	136
C54—H54*A*···O4^iv^	0.99	2.59	3.307 (4)	129
C59—H59*B*···O1	0.99	2.24	3.169 (13)	155
C59*B*—H59*C*···O1*B*	0.99	2.39	3.027 (12)	122

Symmetry codes: (i) −*x*+1, *y*+1/2, −*z*+1; (ii) *x*−1, *y*, *z*; (iii) −*x*+1, *y*−1/2, −*z*+1; (iv) −*x*+1, *y*+1/2, −*z*; (v) −*x*+1, *y*−1/2, −*z*.
